# Vesicular and extravesicular protein analyses from the airspaces of ozone-exposed mice revealed signatures associated with mucoinflammatory lung disease

**DOI:** 10.1038/s41598-021-02256-5

**Published:** 2021-12-01

**Authors:** Ishita Choudhary, Thao Vo, Kshitiz Paudel, Xue Wen, Richa Gupta, Mehmet Kesimer, Sonika Patial, Yogesh Saini

**Affiliations:** 1 Department of Comparative Biomedical Sciences, School of Veterinary Medicine, Louisiana State University, 1909 Skip Bertman Drive, Baton Rouge, LA 70803 USA; 2 Department of Pathobiological Sciences, School of Veterinary Medicine, Louisiana State University, Baton Rouge, LA 70803 USA; 3 Department of Pathology and Laboratory Medicine, UNC School of Medicine, Chapel Hill, NC 27510 USA

**Keywords:** Proteomic analysis, Animal disease models, Respiratory system models, Chronic inflammation

## Abstract

Lung epithelial lining fluid (ELF) harbors a variety of proteins that influence homeostatic and stress responses in the airspaces. Exosomes, nano-sized extracellular vesicles, contain many proteins that vary in abundance and composition based on the prevailing conditions. Ozone causes inflammatory responses in the airspaces of experimental animals and humans. However, the exosomal protein signatures contained within the ELF from ozone-exposed lung airspaces remain poorly characterized. To explore this, we hypothesized that ozone triggers the release of exosome-bound inflammatory proteins from various cells that reflect mucoobstructive lung disease. Accordingly, we repetitively exposed adult male and female C57BL/6 mice to HEPA-filtered air (air) or 0.8 ppm ozone (4 h per day) for 14 days (five consecutive days of exposure, 2 days of rest, five consecutive days of exposure, 2 days of rest, four consecutive days of exposure). Exosome-bound proteomic signatures, as well as the levels of soluble inflammatory mediators in the bronchoalveolar lavage fluid (BALF), were determined 12–16 h after the last exposure. Principal component analyses of the exosome-bound proteome revealed a clear distinction between air-exposed and ozone-exposed mice, as well as between ozone-exposed males and ozone-exposed females. In addition to 575 proteins that were enriched in both sexes upon ozone exposure, 243 and 326 proteins were enriched uniquely in ozone-exposed males and females, respectively. Ingenuity pathway analyses on enriched proteins between ozone- and air-exposed mice revealed enrichment of pro-inflammatory pathways. More specifically, macrophage activation-related proteins were enriched in exosomes from ozone-exposed mice. Cytokine analyses on the BALF revealed elevated levels of G-CSF, KC, IP-10, IL-6, and IL-5 in ozone-exposed mice. Finally, the histopathological assessment revealed significantly enhanced intracellular localization of mucoinflammatory proteins including MUC5B and FIZZ1 in ozone-exposed mice in a cell-specific manner indicating the cellular sources of the proteins that are ferried in the exosomes upon ozone-induced lung injury. Collectively, this study identified exosomal, secretory, and cell-specific proteins and biological pathways following repetitive exposure of mice to ozone.

## Introduction

Exosomes are nano-sized extracellular vesicles (EV) that originate from the endosomal compartment of the cells and are known to contain biomolecules including proteins, lipids, RNA, DNA, and metabolites^1–3^. The composition of these biomolecules in bodily fluids such as plasma^4,5^, epithelial lining fluid (ELF)^6,7^, saliva^8^, milk^8^, and urine^8,9^ may yield valuable information about their cellular origins, physiological roles, prevailing pathological stresses, and, clinically, may have diagnostic and prognostic values^10–12^. All eukaryotic cells release exosomes in healthy as well as stressed conditions and the relative composition of exosomes derived from a variety of cell types lining the body cavity contributes to the overall heterogeneity of the exosome population. Following release into the extracellular milieu, the biological activities of exosomes are mediated either through their direct interaction with the target cells or through their role as a messenger in cell–cell communication^13^. Accordingly, the exosomal cargo proteins can play important roles in the maintenance of tissue homeostasis as well as the manifestation of stress responses.

The epithelial lining fluid (ELF), a thin liquid layer covering the epithelial cells in the airway and alveolar spaces, contains exosomes that are released from the resident (during homeostasis) and/or recruited cells (in stressed conditions)^14^. Encounters between inhaled entities and the cellular and/or molecular constituents of the ELF of the respiratory tract result in the altered composition of the exosome population in the respiratory tract^3,15^. For example, hyperoxia exposure, as well as acid inhalation, results in elevated levels of exosomes in the BALF of mice^16^. However, a detailed proteomic analysis on bronchoalveolar lavage fluid (BALF) exosomes from ozone-exposed mice have never been conducted.

Ozone inhalation causes lung injury and inflammatory responses in experimental animals and humans. In our recent publication^17^, we performed transcriptomic analyses on three distinct lung compartments, i.e., extrapulmonary airways, parenchyma, and purified alveolar macrophages, from mice that were repetitively exposed to filtered air (air) or 0.8 ppm ozone. Here, using the BALF samples from the same set of animals, we investigated whether the enriched protein signatures in BALF reflect upregulation of their transcripts. Accordingly, we hypothesized that repetitive ozone exposure triggers the release of exosome-bound inflammatory proteins from various cells that reflect the mucoobstructive lung disease. Previous studies suggested that, as compared to ozone-exposed males, ozone-exposed females exhibit exaggerated inflammatory responses^17^. Accordingly, our second hypothesis was that exosomes from ozone-exposed females possess unique protein signatures that cause exaggerated inflammatory responses. To test these hypotheses, we asked a series of questions including, (1) Which proteins are enriched in the airspace-derived exosomes from healthy lungs (homeostasis)? (2) Whether the composition and the abundance of exosomal proteins is altered following ozone exposure (stressed environment)? (3) What biological pathways are influenced by the exosomal proteins during homeostasis and under stressed environment? (4) Does the composition of the exosomal proteins reflect the mucoinflammatory disturbances in the airspaces following ozone-induced stress? (5) What are the likely contributors to the key inflammatory proteins in the airspaces following ozone-induced stress? and (6) Whether it is possible to identify the cellular sources of proteins present within the heterogeneous population of airspace exosomes? We attempted to address these questions through comprehensive exosomic-proteomic analyses, immunohistochemical staining, secretory protein measurements, and comparative analyses between proteomic and transcriptomic signatures. This study revealed several interesting findings related to ozone- and sex-specific protein signatures within the pulmonary airspaces.

## Materials and methods

### Animal husbandry

Male and female mice on C57BL/6 background were procured from Jackson Laboratory (Bar Harbor, ME). We used mice on C57BL/6 background because they exhibit robust mucous cell metaplasia and eosinophilic inflammation upon repetitive exposure to ozone^18^. Upon arrival at Louisiana State University (LSU) vivarium, mice were allowed to acclimatize for 3 weeks. Mice were maintained in individually-ventilated, hot-washed cages on a 12 h dark/light cycle. Except during the exposures, mice were maintained on a regular diet and water ad libitum. All methods related to animal experimentation were approved by LSU Institutional Animal Care and Use Committee (IACUC) and performed in accordance with the ethical guidelines and regulations. The authors complied with the Animal Research: Reporting of In Vivo Experiments (ARRIVE) guidelines.

### Experimental design and ozone exposure

Male and female mice, housed in separate cages, were exposed to HEPA-filtered air (air) or HEPA-filtered ozone (806.1 ± 2.68 ppb; 4 h per day) for 14 days (five consecutive days of exposure, two days of rest, five consecutive days of exposure, two days of rest, four consecutive days of exposure). Cages were randomly assigned to the air and ozone exposure groups. We included 14 mice in each of the four experimental groups, i.e., air-exposed males, air-exposed females, ozone-exposed males, and ozone-exposed females. Tissues were harvested from experimental mice 12–16 h after the end of the last exposure. Of note, BALF samples and lung tissues were harvested from the same cohort of mice that were exposed to air or ozone for our recently published study^17^.

Hatch et al. reported that ~ 4–5 times higher inhaled ozone (^18^O_3_) concentration was required for comparable ^18^O labeling of cells and extracellular BALF materials between resting rats and exercising humans^19–21^. The ozone concentration used in this study was ~ 11.5 fold higher than the 8 h National Ambient Air Quality Standards (NAAQS) for ozone, i.e., 0.07 ppm.

Previous reports have highlighted sex-associated differences in the susceptibility to ozone-induced lung injury and inflammation^22–25^; therefore, both sexes were exposed to air or ozone. To replicate real-life exposure conditions of humans during the active phase, mice were exposed in the nightly conditions, a state of higher physical activity in mice^26^. Briefly, the loading of animals onto the light-proof chambers was coordinated with the start of the night cycle at the vivarium. All the exposures took place between 6:00 PM and 11:00 PM.

### Necropsy and tissue harvesting

Mice were anesthetized with an intraperitoneal injection of 2,2,2-tribromoethanol (250 mg/kg; Sigma-Aldrich, St Louis, MO) and thoracotomy was performed to expose lungs and extrapulmonary airways. A 20-gauge cannula was inserted into the trachea and secured in place with a suture. Lungs were lavaged with a calculated volume (Body weight in grams × 0.035 × 1000 = volume in µl) of ice-cold Dulbecco’s phosphate-buffered saline (DPBS) without calcium and magnesium. The first two lavages were pooled and stored on ice. To increase the exosome yield, further lavages were performed to collect additional 9 ml of BALF. Three hundred microliters from the first two lavages were centrifuged at 500*g* for 5 min at 4 °C in order to pellet the cells. Cell-free supernatant was saved at − 80 °C for cytokine analyses. The remaining portion of the first two lavages and 9 ml volume of serial lavages were pooled and centrifuged (as above) to collect cell-free BALF. To increase the exosome yield, cell-free BALF from three individual mice with similar treatment and sex were pooled.

### Exosome isolation from bronchoalveolar lavage fluid (BALF)

Exosomes were isolated by a differential ultracentrifugation method as previously described with some modifications^27^. Briefly, the BALF from three sex- and treatment-matched mice were pooled and centrifuged at 500*g* for 5 min to sediment the BALF immune cells. The cell-free supernatant was further centrifuged at 3000*g* for 10 min to sediment the dead cells. Then, the supernatant was centrifuged at 10,000*g* for 70 min using SW28 rotor (Beckman Coulter Optima L-90 K Ultracentrifuge). The pellet comprising of cell debris and large microvesicles was discarded and the supernatant was filtered using a 0.2 µm filter (VWR, Radnor, PA). The filtered supernatant was further centrifuged at 100,000*g* for 100 min. The supernatant was carefully discarded without disturbing the exosome pellet. The pellet was resuspended in 200 µl of phosphate-buffered saline (PBS). Nanoparticle tracking analyses (NTA; Nanosight 300) on vesicular population harvested using differential ultracentrifugation method typically have a diameter of 131.2 ± 4.4 nm (mean ± SEM) and 102.4 ± 5.8 nm (mode ± SEM). Resuspended exosomes were snap-frozen and stored at − 80 °C. All the centrifugation steps were performed at 4 °C.

### Sample preparation for proteomic analyses

Proteins were reduced, alkylated, and purified by chloroform/methanol extraction prior to digestion with sequencing grade modified porcine trypsin (Promega, Madison, WI). Tryptic peptides were then separated by reverse-phase XSelect CSH C18 2.5 µm resin (Waters) on an in-line 150 × 0.075 mm column using an UltiMate 3000 RSLCnano system (Thermo). Peptides were eluted using a 90 min gradient from 97:3 to 60:40 buffer A:B ratio (Buffer A = 0.1% formic acid, 0.5% acetonitrile; Buffer B = 0.1% formic acid, 99.9% acetonitrile). Eluted peptides were ionized by electrospray (2.15 kV) followed by mass spectrometric (MS) analysis on an Orbitrap Eclipse Tribrid mass spectrometer (Thermo Fisher Scientific, Waltham, MA). MS data were acquired using the FTMS analyzer in profile mode at a resolution of 120,000 over a range of 375 to 1200 m/z. Following HCD activation, MS/MS data were acquired using the ion trap analyzer in centroid mode and normal mass range with a normalized collision energy of 30%.

### Data analysis—intensities

Proteins were identified by database search against the UniprotKB database restricted to *Mus musculus* (November 2019) using MaxQuant (version 1.6.10.43, Max Planck Institute) with a parent ion tolerance of 3 ppm and a fragment ion tolerance of 0.5 Da. Protein identifications were accepted if they could be established with less than 1.0% false discovery and contained at least 2 identified peptides. Protein probabilities were assigned by the Protein Prophet algorithm^28^. Proteins were normalized to iBAQ MS1 intensities within MaxQuant and quality was assessed using the UAMS Bioinformatics core in-house ProteiNorm tool, a user-friendly tool for a systematic evaluation of normalization methods, imputation of missing values, and comparisons of different differential abundance methods. Popular normalization methods are evaluated including log2 normalization (Log2), median normalization (Median), mean normalization (Mean), variance stabilizing normalization (VSN)^29^, quantile normalization (Quantile)^30^, cyclic loess normalization (Cyclic Loess)^31^, global robust linear regression normalization (RLR)^32^, and global intensity normalization (Global Intensity)^32^. The individual performance of each method can be evaluated by comparing the following metrices: total intensity, Pooled intragroup Coefficient of Variation (PCV), Pooled intragroup Median Absolute Deviation (PMAD), Pooled intragroup estimate of variance (PEV), intragroup correlation, sample correlation heatmap (Pearson), and log2-ratio distributions. The data were normalized using VSN as this method had the lowest intragroup variance and highest intragroup correlation. The Log2 VSN normalized iBAQ MS1 intensities were used to perform statistical analysis using Linear Models for Microarray Data (limma) with empirical Bayes (eBayes) smoothing to the standard errors^31^. Proteins with an adjusted *p*-value < 0.05 and a fold change (FC) > 2 were considered to be significant. Significant proteins were used to identify important protein networks and pathways using the Ensemble of Gene Set Enrichment Analyses (EGSEA) Bioconductor package^33^.

### Ingenuity pathway and protein interaction networks analyses

The Ingenuity pathway analysis (IPA) identifies canonical pathways and biological networks that are activated in the queried data set. The differentially enriched proteins in ozone-exposed mice were subjected to IPA to investigate the biological networks and pathways that were enriched in the exosomes (Qiagen, Redwood City, CA). Core analysis function was selected to screen proteins that met our cutoff criteria (FC > 2, FDR < 0.05). STRING analysis was performed to identify protein–protein interaction network enrichment in enriched proteins^34^. The STRING (https://string-db.org; version 11.0) maintains a database of known and predicted protein–protein interaction (PPI) networks.

### Analyses of BALF for cytokines

Cell-free BALF was assessed for granulocyte-colony stimulating factor (G-CSF), keratinocyte chemoattractant (KC/CXCL1), IP-10, IL-6, and IL-5 using a Luminex XMAP-based assay (MCYTOMAG-70 K), according to manufacturer’s instructions (EMD Millipore, Billerica, MA).

### Immunohistochemistry for MUC5B and RETNLA (FIZZ1/RELMα)

A separate cohort of mice was exposed to air or ozone to harvest unlavaged lung lobes for histopathological tissue preparation. Lungs were inflated with a calculated volume (Body weight in grams × 0.035 × 1000 = volume in µl) of 10% neutral buffered formalin. Formalin-fixed left lung lobes were transversally sectioned twice along the main stem bronchus, i.e., at the hilum and 2 mm caudally to the hilum. Sections were paraffin-embedded and 5 µm thick sections were mounted onto glass slides. Sections were processed for immunohistochemical staining, as previously described.^35,36^ Lung sections were processed to assess immunohistochemical localization of MUC5B, as previously described^35,36^. Rabbit monoclonal RETNLA (FIZZ1) primary antibody (ab39626; ABCAM Cambridge, MA) was used to probe lung sections. To quantify MUC5B/RETNLA stained cells, the number of MUC5B/RETNLA stained cells and the total number of cells in the airways were manually counted.

### Statistical analyses

Data were analyzed using Shapiro Wilk’s and Levene’s test for the determination of normality and equality of variances, respectively. If any one of these assumptions, that are critical for the application of parametric ANOVA, were violated, non-parametric Kruskal Wallis ANOVA was used to detect the significant differences in the main effect. In case of significant main effect, Dunn’s post hoc test was used for multiple comparisons to determine the significant differences among groups. All data were expressed as mean ± standard deviation (SD). Outliers were eliminated by using Grubb’s test application. Statistical analyses were performed using GraphPad Prism 9.0 (GraphPad Software, La Jolla, CA). A *p*-value of less than 0.05 was considered statistically significant.

## Results

### Ozone exposure results in exaggerated lung injury and a robust increase in BALF cytokine levels

**Figure 1:**
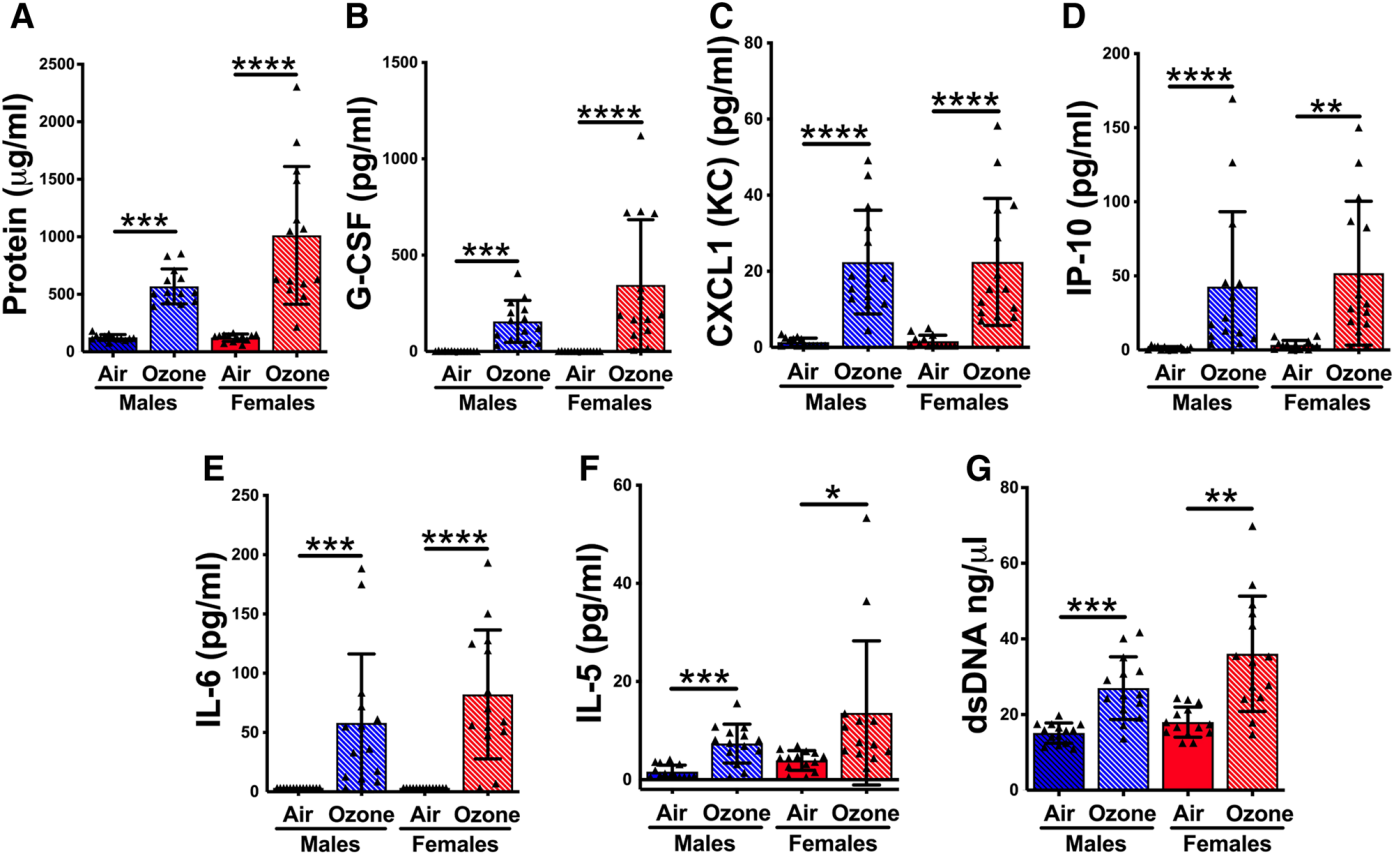
(**A**) Ozone exposure causes exaggerated lung injury and inflammatory mediators in ozone-exposed mice. (**A**) Protein concentration (µg/ml) in the cell-free BALF from the air- and ozone-exposed males and females. Significant main effect was detected (Kruskal Wallis Statistic = 40.34; df = 3; *p* < 0.0001). Concentration (pg/ml) of G-CSF (**B**), CXCL1 (KC) (**C**), IP-10 (**D**), IL-6 (**E**), and IL-5 (**F**), in the cell-free BALF from air- and ozone-exposed males and females. The G-CSF and IL-6 concentrations for air-exposed groups in Panels B and E, respectively, were below detection limit. The values for such samples were obtained by subtracting 0.01 from lowest detection limit. Significant main effect was detected for G-CSF, KC, IP-10, IL-6, and IL-5 (Kruskal Wallis Statistic = 35.67 for IL-6, 41.72 for G-CSF, 27.64 for IL-5, 35.97 for IP-10, and 41.15 for KC; df = 3 for all; *p* < 0.0001 for all). (**G**) Double-stranded DNA (dsDNA) concentration (ng/ml) in the cell-free BALF from the air- and ozone-exposed males and females. Significant main effect was detected (Kruskal Wallis Statistic = 29.89; df = 3; *p* < 0.0001). Error bars represent Standard Deviation (SD). *p < 0.05, **p < 0.01, ***p < 0.001, ****p < 0.0001 using Kruskal Wallis test followed by Dunn’s post hoc comparisons. (n = 13–14 per group). BALF, bronchoalveolar lavage fluid; G-CSF, Granulocyte colony-stimulating factor; CXCL1, Chemokine (C-X-C motif) ligand 1; KC, Keratinocytes-derived chemokine; IP-10, Interferon-gamma induced protein 10; IL-6, Interleukin 6; IL-5, Interleukin 5.

To assess lung injury in response to ozone, we analyzed the total protein contents of cell-free BALF, an indicator of alveolar-endothelial gas exchange barrier damage^37,38^. While the BALF protein contents in air-exposed mice were comparable between males (122.5 ± 8.9 µg/ml) and females (124.3 ± 7.1 µg/ml), both ozone-exposed males (568.8 ± 42.5 µg/ml) and females (1012.0 ± 159.9 µg/ml) had significantly elevated protein contents (Fig. 1A). Further, BALF protein contents in ozone-exposed females trended higher than those in ozone-exposed males (Fig. 1A).

To determine the levels of soluble inflammatory mediators in the airspaces, we assessed levels of 25 cytokines in the BALF from the air- and the ozone-exposed mice. Only five of the analyzed cytokines showed a significant main effect (Fig. 1B–F). While air-exposed males and females had basal or undetectable levels of all these five cytokines, ozone exposure resulted in a significant elevation in the concentration of these five cytokines, i.e., G-CSF, KC, IP-10, IL-6, and IL-5 in both the sexes (Fig. 1B–F). No significant difference was observed between ozone-exposed males versus ozone-exposed females for any of the five cytokines.

Next, we assessed the concentration of double-stranded DNA (dsDNA), an indicator of cellular injury associated with neutrophilic inflammation^39–41^. BALF dsDNA levels were comparable between air-exposed males and air-exposed females (Fig. 1G). BALF from both ozone-exposed males and ozone-exposed females had significantly elevated levels of dsDNA as compared to the respective control groups. The dsDNA levels trended higher in ozone-exposed females versus ozone-exposed males (Fig. 1G).

### Isolation of BALF exosomes and assessment for exosome-specific markers

**Figure 2:**
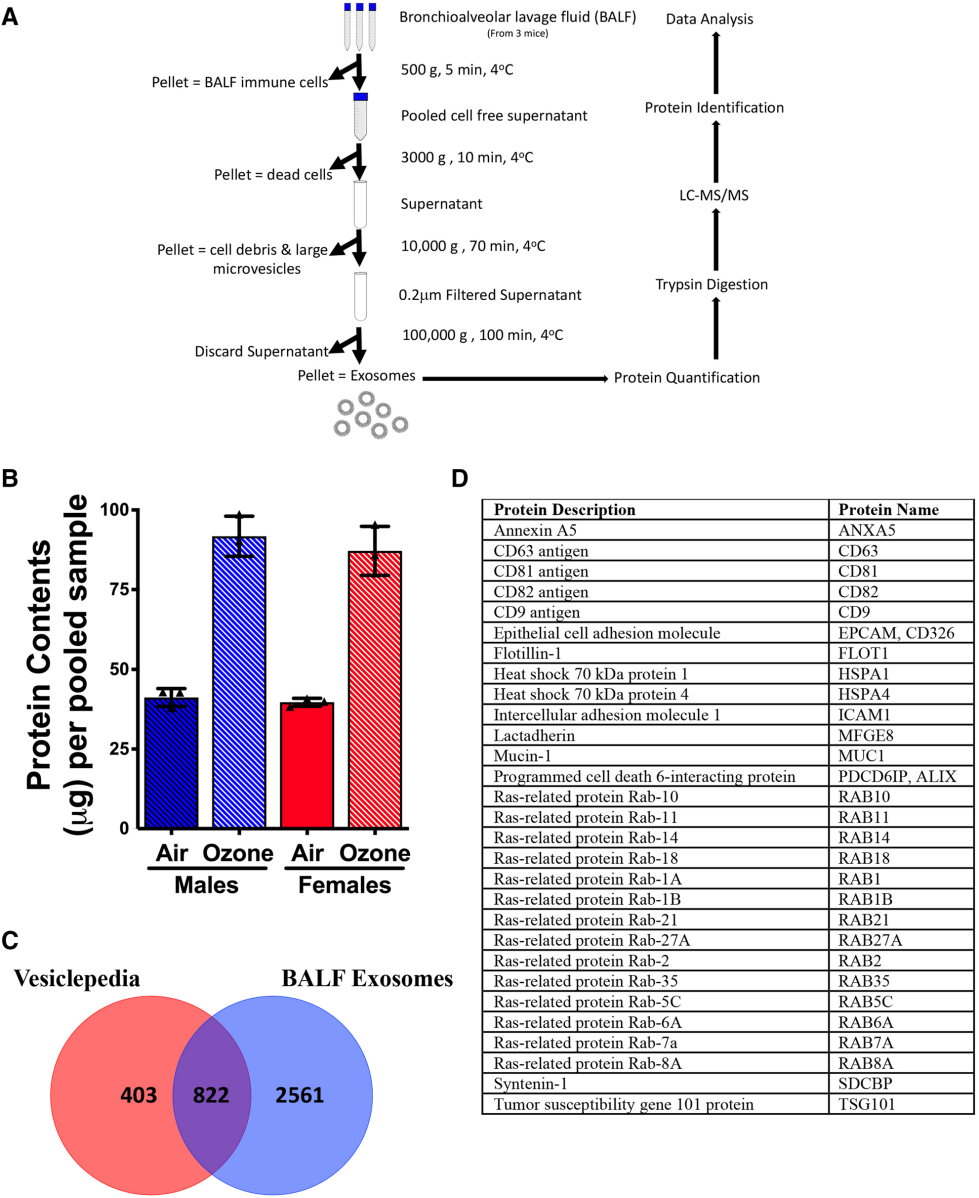
Exosome harvest and analyses for exosome-specific protein signatures. (**A**) Flow diagram (created with www.biorender.com) delineating designated steps involved in BALF processing for proteomics data analyses. (**B**) Total protein yield (µg) in the exosomes harvested from the air- and ozone-exposed males and females. Significant main effect was detected (Kruskal Wallis Statistic = 8.744; df = 3; *p* = 0.006). Error bars represent Standard Deviation (SD). Analysis was done using Kruskal Wallis test followed by Dunn’s post hoc comparisons (n = 3 per group). (**C**) Venn diagram to show that 822 out of 1225 exosome-specific markers obtained from Vesiclepedia database were present in exosomes harvested in this study. (**D**) Table showing 29 exosome markers that were represented in the top 20% of the most abundant proteins.

Cell-free BALF was subjected to differential centrifugation to sediment exosomes (Fig. 2A). The total protein contents in the exosomes from air-exposed males (41.2 ± 1.6 µg) were comparable with air-exposed females (39.7 ± 0.7 µg). The total protein contents in the exosomes from ozone-exposed mice trended higher as compared to air-exposed mice (Fig. 2B). The protein contents were comparable between ozone-exposed male (91.7 ± 3.6 µg) and ozone-exposed female (87.2 ± 4.4 µg) mice (Fig. 2B**)**.

Next, a list of 1225 exosomal protein signatures was retrieved from the Exocarta Vesiclepedia database. This list contained proteins that fulfilled designated criteria (*Species*-Mus Musculus; *Tissues/cell type*-Lung cells, macrophages, mast cells, fibroblast, BALF, B cells, Plasma, Serum and thymus; *Cell line*-dendritic cells, macrophages, mast cells, myeloid-derived suppressor cells, and T-cells; *Isolation methods*-all reported; *Detection methods*-ELISA, Mass spectrophotometry, immunoelectron microscopy, and western blotting; *Vesicle types*-Exosomes). Out of 1225 exosomal protein signatures, 822 were identified in BALF exosomes collected in this study (Fig. 2C). Next, through a manual literature search, we generated a list of 50 proteins that are known to be exosome-specific signatures. While all of these 50 known exosome markers were present in all the 12 exosome samples, at least 29 of these proteins were represented in the top 20% of the most abundant proteins (Fig. 2D).

### Exosomes from filtered air-exposed mice contain lung cell-specific proteins

A total of 3258 proteins were identified in exosomes from air-exposed mice. While 2361 proteins were present in all six (3 males; 3 females) exosome samples from air-exposed mice, 320, 185, 137, 129, and 126 additional proteins were identified in at least 5, 4, 3, 2, and 1 exosome samples from air-exposed mice, respectively. A list of 50 most-abundant proteins in exosomes from air-exposed mice is included in Table 1. Our analyses revealed that exosomes from air-exposed mice contain protein signatures known to be expressed in the lungs including club cell-specific protein (SCGB1A1; uteroglobin; CCSP), surfactant-associated protein D (SFTPD), surfactant-associated protein B (SFTPB), surfactant-associated protein A1 (SFTPA1), chitinase-like protein 3 (CHIL3), chitinase-3-like protein 1 (CHI3L1), alpha-1-antitrypsin 1–2 (SERPINA1B), serotransferrin (TF), superoxide dismutase (SOD1), and platelet glycoprotein 4 (CD36). The presence of these proteins suggests that; 1) the exosome populations are representative of the homeostatic airspaces and, 2) the proteins detected within the exosomes may have potential roles in immune defense and antioxidant responses.

**Table 1:** Top 50 protein signatures enriched in air- and ozone-exposed mice.

Exosomal proteins enriched in air-exposed mice				Exosomal proteins enriched in ozone-exposed mice			
Protein	Description	Ranking in		Protein	Description	Ranking in	
		Air	Ozone			Ozone	Air
ALB	Serum albumin	1	1	ALB	Serum albumin	1	1
SCGB1A1	Uteroglobin	2	3	HIST1H4A	Histone H4	2	**126**
TF	Serotransferrin	3	8	SCGB1A1	Uteroglobin	3	2
SFTPD	Pulmonary surfactant-associated protein D	4	5	HIST1H2B	Histone H2B	4	**324**
TTR	Transthyretin	5	12	SFTPD	Pulmonary surfactant-associated protein D	5	4
SFTPA1	Pulmonary surfactant-associated protein A	6	9	ACTB	Actin, cytoplasmic 1	6	9
CYP2F2	Cytochrome P450 2F2	7	36	HIST1H2A	Histone H2A	7	**381**
CES1D	Carboxylesterase 1D	8	29	TF	Serotransferrin	8	3
ACTB	Actin, cytoplasmic 1	9	6	SFTPA1	Pulmonary surfactant-associated protein A	9	6
PRDX6	Peroxiredoxin-6	10	11	H3F3A	Histone H3.2;Histone H3	10	**376**
PRSS1	Cationic Trypsinogen	11	26	PRDX6	Peroxiredoxin-6	11	10
AHSG	Alpha-2-HS-glycoprotein	12	16	TTR	Transthyretin	12	5
HP	Haptoglobin	13	20	TRY4;TRY5	Trypsin 4; 5	13	**72**
HPX	Hemopexin	14	24	GPRC5A	Retinoic acid-induced protein 3	14	29
LYZ2	Lysozyme C-2	15	**82**	BPIFB1	BPI fold-containing family B member 1	15	20
ALDH1A1	Retinal dehydrogenase 1	16	22	AHSG	Alpha-2-HS-glycoprotein	16	12
CHIL3	Chitinase-like protein 3	17	**67**	ANXA5	Annexin A5	17	36
CYB5A	Cytochrome b5	18	**53**	RPS27A	Ubiquitin-40S ribosomal protein S27a	18	40
SERPINA1E	Alpha-1-antitrypsin 1–5	19	34	APOA4	Apolipoprotein A-IV	19	27
BPIFB1	BPI fold-containing family B member 1	20	15	HP	Haptoglobin	20	13
HBA	Hemoglobin subunit alpha	21	39	HIST1H1C	Histone H1.2	21	**382**
SOD1	Superoxide dismutase [Cu–Zn]	22	41	ALDH1A1	Retinal dehydrogenase 1	22	16
CHI3L1	Chitinase-3-like protein 1	23	**109**	SEC14L3	SEC14 like Lipid Binding 3	23	32
SELENBP1;2	Selenium-binding protein 1; 2	24	27	HPX	Hemopexin	24	14
SERPINA1D	Alpha-1-antitrypsin 1–4	25	25	SERPINA1D	Alpha-1-antitrypsin 1–4	25	25
CES1C	Carboxylesterase 1C	26	50	PRSS1	Cationic Trypsinogen	26	11
APOA4	Apolipoprotein A-IV	27	19	SELENBP1;2	Selenium-binding protein 1; 2	27	24
HBB-BS	Hemoglobin subunit beta-1	28	**54**	GSN	Gelsolin	28	37
GPRC5A	Retinoic acid-induced protein 3	29	14	CES1D	Carboxylesterase 1D	29	8
PON1	Serum paraoxonase/arylesterase 1	30	**104**	ANXA1	Annexin A1	30	**102**
SFTPB	Pulmonary surfactant-associated protein B	31	**78**	MSN	Moesin	31	39
SEC14L3	SEC14 like Lipid Binding 3	32	23	CALM1	Calmodulin 1	32	48
METTL7A1	Methyltransferase-like protein 7A	33	**144**	ANXA2	Annexin A2;Annexin	33	**70**
POR	NADPH–cytochrome P450 reductase	34	**103**	SERPINA1E	Alpha-1-antitrypsin 1–5	34	19
SERPINA1B	Alpha-1-antitrypsin 1–2	35	**76**	CD36	Platelet glycoprotein 4	35	44
ANXA5	Annexin A5	36	17	CYP2F2	Cytochrome P450 2F2	36	7
GSN	Gelsolin	37	28	ANXA3	Annexin A3	37	**63**
CBR2	Carbonyl reductase [NADPH] 2	38	40	SDCBP	Syntenin-1	38	**113**
MSN	Moesin	39	31	HBA	Hemoglobin subunit alpha	39	21
RPS27A	Ubiquitin-40S ribosomal protein S27a	40	18	CBR2	Carbonyl reductase [NADPH] 2	40	38
FTL1;FTL2	Ferritin	41	**83**	SOD1	Superoxide dismutase [Cu–Zn]	41	22
MGST1	Microsomal glutathione S-transferase 1	42	**131**	APOA1	Apolipoprotein A-I	42	**91**
FTH1	Ferritin heavy chain	43	**66**	AQP5	Aquaporin-5	43	**79**
CD36	Platelet glycoprotein 4	44	35	S100A11	Protein S100-A11	44	**110**
IGHG2B	Ig gamma-2B chain C region	45	**101**	S100A6	Protein S100-A6	45	**96**
P4HB	Protein disulfide-isomerase	46	**141**	RETNLA	Resistin-like alpha	46	**367**
TUBB4B; 4A	Tubulin beta-4B chain; 4A chain	47	47	TUBB4B;4A	Tubulin beta-4B chain; 4A chain	47	47
CALM1	Calmodulin 1	48	32	HSPA8	Heat shock cognate 71 kDa protein	48	**82**
SERPINC1	Antithrombin-III	49	**84**	RHOA	Transforming protein RhoA	49	**68**
C5	Complement C5	50	**151**	CES1C	Carboxylesterase 1C	50	26

Ranking column on the right of Air (or Ozone) column indicates the ranking of the protein in the ozone (or air) group. Bold text indicate that the corresponding protein is not present in top 50 in the respective exposure group.

**Figure 3:**
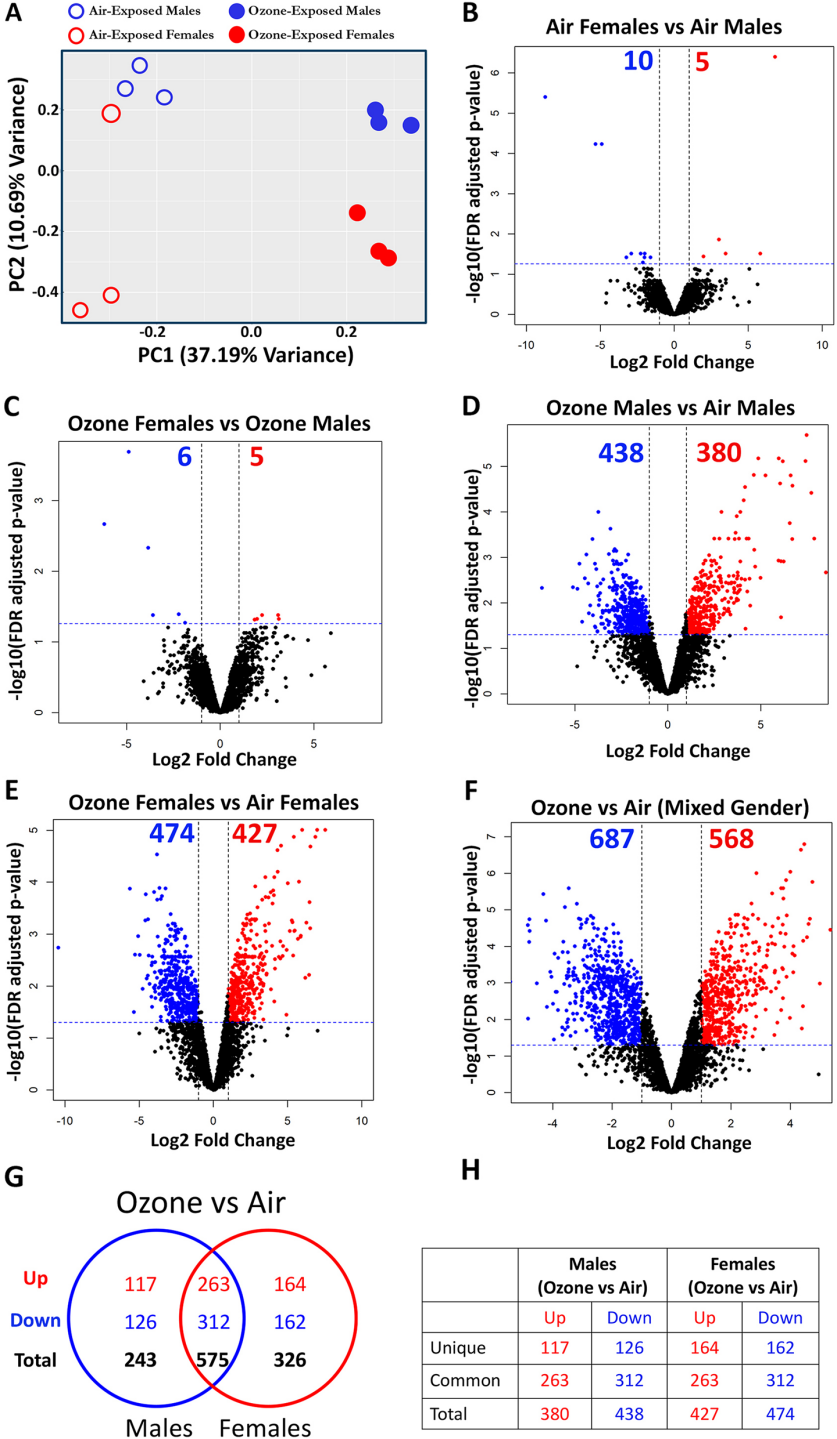
Ozone exposure results in alterations in the airspace exosome-bound proteome. (**A**) Two-dimensional principal component (PC) analysis plot using PC1 and PC2 on differentially enriched proteins (after normalization) in exosomes from the air- and ozone-exposed mice. (**B–F**) Volcano plots depicting differentially abundant proteins (enriched and low abundance) in four different comparisons that were identified using cutoff criteria [Log2 Fold change > 2, -Log10 (FDR adjusted *p*-values < 0.05)]. (**B**) air-exposed females versus air-exposed males, (**C**) ozone-exposed females versus ozone-exposed males, (**D**) ozone-exposed males versus air-exposed males, (**E**) ozone-exposed females versus air-exposed females. (n = 3 per sex per treatment) and (**F**) ozone-exposed mice (both sexes) versus air-exposed mice (both sexes). Venn diagram (**G**) depicting common and unique differentially enriched proteins (enriched and low abundance) in ozone-exposed males versus air-exposed males and ozone-exposed females versus air-exposed females. Tabular (**H**) summary of the Venn diagram.

Principal component (PC) analyses revealed clear clustering of air-exposed male samples, but the air-exposed female samples were somewhat dispersed (Fig. 3A). Next, employing stringent cutoff criteria (Log2 Fold change > 1, FDR < 0.05), we compared differentially enriched protein signatures in air-exposed females versus air-exposed males (Table 2A and Fig. 3B). Our analyses identified only 15 differentially enriched (5 upregulated and 10 downregulated) proteins (Table 2A and Fig. 3B).

**Table 2:** Top differentially enriched protein signatures between (A) air-exposed females and air-exposed males, (B) ozone-exposed females and ozone-exposed males.

Protein	Description	FC	Adj. *p*. val
(A) Air-exposed females vs air-exposed males			
**Upregulated**			
SIPA1L3	Signal-induced proliferation-associated 1-like protein 3	112.60	4.0E−07
CDCP1	CUB domain-containing protein 1	56.22	0.0306
HECTD1	E3 ubiquitin-protein ligase HECTD1	11.09	0.0306
CGN	Cingulin	8.07	0.0137
NA	Ig kappa chain V–III region PC 3741/TEPC 111; Ig kappa chain V–III region TEPC 124	3.92	0.0361
**Downregulated**			
PTPN23	Tyrosine-protein phosphatase non-receptor type 23	− 424.61	0.0000
MUP20	Major urinary protein 20	− 40.28	0.0001
MUP4, 6, 8, 9, 19	Major urinary protein 4, 6, 8, 9, 19	− 29.90	0.0001
CLIP1	CAP-Gly domain-containing linker protein 1	− 9.45	0.0383
WNK1	Serine/threonine-protein kinase WNK1	− 7.51	0.0306
MUP10; MUP1	Major urinary protein 10	− 4.84	0.0306
C8G	Complement component C8 gamma chain	− 4.34	0.0511
CFL2	Cofilin-2	− 4.07	0.0383
SERPINA1E	Alpha-1-antitrypsin 1–5	− 3.99	0.0306
CDK16, 17, 18	Cyclin-dependent kinase 16, 17, 18	− 3.06	0.0383
(B) Ozone-exposed females vs ozone-exposed males			
**Upregulated**			
CD5L	CD5 antigen-like	8.75	0.0471
VAMP5	Vesicle-associated membrane protein 5	8.51	0.0417
MSLN	Mesothelin; Megakaryocyte-potentiating factor; Mesothelin, cleaved form	4.72	0.0417
NA	Ig kappa chain V-III region PC 3741/TEPC 111; Ig kappa chain V-III region TEPC 124	3.89	0.0471
LTF	Lactotransferrin	3.56	0.0482
**Downregulated**			
H3F3C; H3F3A	Histone H3.3C;Histone H3.3;Histone H3	− 73.52	0.0021
MUP4; MUP9; MUP6;MUP8; MUP19	Major urinary protein 6; Major urinary proteins 11 and 8	− 29.65	0.0002
MUP20	Major urinary protein 20	− 14.52	0.0046
CFAP20	Cilia- and flagella-associated protein 20	− 12.13	0.0417
PRKAR2B	cAMP-dependent protein kinase type II-beta regulatory subunit	− 4.66	0.0404
AKAP2; PAKAP	A-kinase anchor protein 2	− 3.71	0.0533

### Exosomes from ozone-exposed mice contain unique protein signatures relevant to lung inflammation

A total of 3421 proteins were identified in exosomes from ozone-exposed mice. A total of 2756 proteins were present in all six exosome samples from ozone-exposed mice. 288, 139, 108, 75, and 55 proteins were identified in at least 5, 4, 3, 2, and 1 exosome samples from ozone-exposed mice, respectively. A list of 50 most-abundant proteins in exosomes from ozone-exposed mice is included in Table 1. Comparison of lists indicating 50 most-abundant proteins in exosomes from air-exposed and ozone-exposed mice identified 33 common signatures and 17 treatment-specific signatures. Most abundant proteins from the top 50 in exosomes that were specific to ozone treatment included RETNLA, AQP5, HSPA, S100A6, S100A11, HIST1H4A, HIST1H1C, HIST1H2A’s, HIST1H2B’s, HIST1H3B, and APOA1, a majority of which are known lung inflammatory proteins.

PC analyses revealed a clear separation of ozone-exposed female and ozone-exposed male samples. The separation was contributed by PC2 that accounts for ~ 11% variance (Fig. 3A). Next, we compared differentially enriched protein signatures in ozone-exposed females and ozone-exposed males (Table 2B and Fig. 3C). Our analyses identified an enrichment of 5 proteins and a reduced abundance of 6 proteins (Table 2B and Fig. 3C).

Of note, we found additional differentially enriched proteins that were eliminated from the analyses because those proteins were not detected in one of the groups being compared and were assigned NA designation during the normalization process. In total, 22 such proteins were exclusively present in air-exposed females (Supplemental Table 1A) and 56 such proteins were exclusively present in air-exposed males (Supplemental Table 1B). Further, 23 proteins were exclusively present in ozone-exposed females (Supplemental Table 2A) and 53 proteins were exclusively present in ozone-exposed males (Supplemental Table 2B). Finally, 27 and 168 proteins were exclusively present in the air- and ozone-exposed mice, respectively (Supplemental Table 3).

**Table 3:** Top 50 protein signatures that were enriched in ozone-exposed mice versus air-exposed mice.

Common in both sexes					Unique to males			Unique to females		
Protein	FC (Males)	Adj. *p*. val (Males)	FC (Females)	Adj. *p*. val (Females)	Protein	FC	Adj. *p*. val	Protein	FC	Adj. *p*. val
H1F0	366.00	0.0021	87.75	0.0061	CDCP1	68.53	0.0012	PTPN23	185.25	9.81E−06
EPHA2	235.79	0.0004	89.75	0.0002	SIPA1L3	29.20	0.0000	CD151	59.59	0.0011
H3F3A	212.11	0.0000	4.77	0.0268	ITGB6	21.11	0.0029	CHMP2A	30.31	0.0356
HIST1H1B	177.60	0.0000	43.06	0.0000	STX4	18.14	0.0369	APOD	17.85	0.0030
H2AFJ	169.82	0.0000	117.94	0.0000	PRPF8	13.15	0.0085	DLG1	17.29	0.0197
HIST1H1E	104.76	0.0000	54.44	0.0001	SNRPN	13.01	0.0089	SLC6A6	14.66	0.0115
H2AFY	103.52	0.0004	43.86	0.0009	ARFGEF2	11.93	0.0059	CKMT1	12.03	0.0057
HIST1H2BR	97.76	0.0000	92.89	0.0000	HNRNPA1	11.68	0.0100	PLA2G7	11.14	0.0038
HIST1H3A	95.05	0.0002	77.01	0.0006	HSPA2	10.71	0.0036	CHMP1B	10.32	0.0094
GP2	75.59	0.0012	58.81	0.0011	TARDBP	10.36	0.0181	RPL35A	10.28	0.0437
HIST1H3B	72.74	0.0000	127.66	0.0000	PTGS2	10.11	0.0523	PLAUR	10.26	0.0099
TNC	68.46	0.0206	73.58	0.0069	TRIM28	9.71	0.0037	GLUD1	9.71	0.0127
HIST1H1C	65.82	0.0000	32.16	0.0001	FBL	9.16	0.0055	MMP10	9.60	0.0214
HIST1H4A	62.93	0.0000	63.08	0.0000	NCL	8.12	0.0168	MYO18A	9.08	0.0038
CKAP5	62.45	0.0012	41.38	0.0026	LSR	7.67	0.0044	PIP5K1A	8.93	0.0075
HIST1H1D	38.31	0.0000	16.95	0.0002	RBBP7	7.58	0.0087	RFTN1	8.78	0.0271
SDCBP2	31.76	0.0028	51.70	0.0012	COL4A3BP	7.24	0.0029	TSPO	8.71	0.0164
SNRPD1	25.93	0.0016	22.95	0.0014	EIF2B5	7.18	0.0033	CHMP5	7.08	0.0117
EFTUD2	25.11	0.0007	25.33	0.0026	MAP4	6.55	0.0047	RALBP1	6.80	0.0408
LLGL2	24.69	0.0000	19.85	0.0000	LRRC8C	5.94	0.0061	DYNLT3	6.71	0.0338
SLC23A2	21.64	0.0114	92.30	0.0008	KEAP1	5.66	0.0314	GM4788	6.51	0.0118
SLC26A4	20.33	0.0004	8.83	0.0017	PPFIBP2	5.63	0.0230	CPNE1	6.50	0.0038
MATN4	19.45	0.0056	8.35	0.0044	STUB1	5.61	0.0122	SH3GL1	6.46	0.0027
EPB41L5	18.99	0.0004	11.32	0.0009	NUMB	5.46	0.0197	PKP3	6.35	0.0094
ITGAV	17.78	0.0000	23.53	0.0000	EPB41L4B	5.44	0.0176	CYBB	6.29	0.0250
KRT8	16.91	0.0001	9.81	0.0002	APPL2	5.16	0.0287	HADH	6.29	0.0117
HP1BP3	15.64	0.0029	11.95	0.0061	ANXA8	5.12	0.0250	COPS6	6.14	0.0085
ITGA3	14.91	0.0001	17.14	0.0001	SEPTIN_9	5.02	0.0114	CAV2	6.11	0.0065
ABHD4	14.24	0.0004	13.06	0.0002	USP4	4.99	0.0121	PAPSS2	6.09	0.0324
COL6A2	13.71	0.0086	22.75	0.0029	CGN	4.92	0.0045	ARFGEF1	5.96	0.0090
CPNE8	13.68	0.0042	40.43	0.0006	STEAP4	4.79	0.0147	MLLT4	5.95	0.0079
DDX5	13.59	0.0025	9.18	0.0036	PRMT1	4.64	0.0089	EXOC8	5.92	0.0065
ITGA6	13.47	0.0021	11.89	0.0022	GGA1	4.44	0.0118	CAV1	5.81	0.0069
RETNLA	13.22	0.0029	15.46	0.0017	RBBP4	4.38	0.0397	CARS	5.75	0.0532
POSTN	13.11	0.0080	21.36	0.0027	HECTD1	4.31	0.0206	SLC39A8	5.56	0.0085
CTPS1	13.08	0.0001	16.39	0.0001	DNM3	4.22	0.0291	MSLN	5.55	0.0013
DHX9	13.04	0.0089	9.61	0.0120	OSMR	4.17	0.0533	SORBS3	5.50	0.0197
LMNA	12.51	0.0003	14.49	0.0002	EXOC4	4.15	0.0134	COL4A2	5.46	0.0140
NDNF	12.00	0.0036	30.60	0.0020	ARF5	4.15	0.0478	Q8CEZ4	5.39	0.0205
MACF1	11.87	0.0047	38.90	0.0005	BRCC3	4.12	0.0279	XPNPEP1	5.20	0.0291
S100A16	11.60	0.0025	7.81	0.0042	SUGT1	3.99	0.0280	SNX4	5.17	0.0176
CAPN7	10.88	0.0036	10.04	0.0030	DNAJB4	3.85	0.0530	RAB11FIP1	5.14	0.0095
MUC5AC	9.84	0.0156	17.73	0.0046	RASGRF2	3.84	0.0208	COPS8	5.08	0.0200
FN1	9.58	0.0004	11.42	0.0002	HERC4	3.81	0.0145	VPS29	5.06	0.0441
CHMP3	8.72	0.0036	11.83	0.0014	RTKN	3.78	0.0429	CLIP1	4.94	0.0142
PLXNA1	7.22	0.0010	20.10	0.0001	ATG7	3.74	0.0419	AGO2	4.80	0.0224
COL6A3	7.04	0.0025	15.77	0.0003	ANKRD13A	3.64	0.0510	EIF3E	4.60	0.0114
PTPRE	6.92	0.0211	26.01	0.0017	KLC2	3.63	0.0427	GIPC1	4.53	0.0056
BIRC6	6.53	0.0263	44.62	0.0008	NEK9	3.56	0.0540	MON2	4.47	0.0062
ZDHHC5	5.96	0.0139	35.61	0.0009	LPP	3.48	0.0197	CRYAB	4.42	0.0026

### Exosomes from ozone-exposed mice are enriched in stress-response proteins in a sex-specific manner

The top two principal components (PC1 and PC2), that contribute to ~ 48% variance, revealed that treatment and sex were the primary drivers of variation in overall protein contents. The PC1, which accounts for 37.19% of the variance, separated air-exposed mice from ozone-exposed mice. PC2, which accounts for 10.69% of overall variance, distinctly separated ozone-exposed males and ozone-exposed females (Fig. 3A). Next, a comparison of ozone-exposed males and air-exposed males identified 818 differentially expressed (380, enriched; 438, low abundance) proteins (Fig. 3D and Supplemental Table 4). Similarly, a comparison of ozone-exposed females and air-exposed females identified 901 differentially expressed (427, enriched; 474, low abundance) proteins (Fig. 3E and Supplemental Table 4). Using cutoff criteria (Log2FC > 1, FDR < 0.05), a comparison of ozone-exposed mice (mixed sex) with air-exposed mice (mixed sex) identified 1255 differentially expressed (568, enriched; 687, low abundance) proteins (Fig. 3F and Supplemental Table 4).

**Table 4:** Top 50 protein signatures that had significantly reduced abundance in ozone-exposed mice versus air-exposed mice.

Common in both sexes					Unique to males			Unique to females		
Protein	FC (Males)	Adj. *p*. val (Males)	FC (Females)	Adj. *p*. val (Females)	Protein	FC	Adj. *p*. val	Protein	FC	Adj. *p*. val
MLF1	− 109.96	0.0047	− 1407.91	0.0018	FABP1	− 34.74	0.00454	E030010N08RIK	− 41.09	0.0315
GDPD1	− 29.31	0.0049	− 9.27	0.0250	SLC22A18	− 24.44	0.02592	ATP13A1	− 27.45	0.0113
PMPCA	− 27.02	0.0014	− 31.38	0.0025	MAGT1	− 19.03	0.01691	ARHGEF7	− 22.66	0.0071
SLC27A2	− 22.15	0.0029	− 33.84	0.0011	UFL1	− 16.41	0.01862	RNF213	− 21.56	0.0063
MERTK	− 20.86	0.0009	− 6.88	0.0031	AFP	− 15.18	0.03291	DAD1	− 19.74	0.0090
EFHC1	− 19.61	0.0037	− 6.09	0.0261	EMD	− 14.99	0.03432	MIA3	− 18.24	0.0161
CLCC1	− 18.19	0.0114	− 15.72	0.0112	DNAH9	− 13.86	0.04258	GPAA1	− 17.64	0.0250
CYP4A12	− 16.69	0.0004	− 23.68	0.0002	IGHV1-47	− 13.82	0.04187	SLC27A1	− 15.49	0.0054
FGFR2	− 15.19	0.0014	− 49.82	0.0001	FKBP8	− 13.19	0.01126	ITPR3	− 15.14	0.0129
GM2A	− 14.42	0.0018	− 24.17	0.0005	FAM3C	− 12.90	0.0085	LMF1	− 13.03	0.0107
MTDH	− 14.01	0.0043	− 11.51	0.0051	CES2	− 12.05	0.02864	KDELC2	− 12.61	0.0142
CYP4F	− 13.44	0.0070	− 17.41	0.0035	RILPL1	− 11.17	0.00304	THEM6	− 12.54	0.0105
LSP1	− 13.42	0.0001	− 9.36	0.0001	IGKV9-124	− 9.75	0.0236	EMC4	− 11.74	0.0461
IYD	− 12.55	0.0061	− 9.62	0.0085	HM13	− 9.05	0.04311	NBAS	− 11.52	0.0093
BNIP1	− 12.28	0.0037	− 12.61	0.0017	SPCS3	− 9.04	0.02631	BICD2	− 11.34	0.0170
MLF2	− 12.01	0.0206	− 11.17	0.0083	PCCB	− 8.82	0.00454	SPAG6	− 10.84	0.0130
TRP53I11	− 11.40	0.0148	− 11.64	0.0183	ALG2	− 7.90	0.04321	LCLAT1	− 10.56	0.0144
CRELD2	− 11.30	0.0165	− 11.72	0.0081	PCCA	− 7.87	0.00526	CNPY2	− 10.35	0.0075
ILVBL	− 10.50	0.0357	− 8.61	0.0465	CELA1	− 7.78	0.05386	GNN	− 10.27	0.0036
BC017158	− 10.19	0.0147	− 12.11	0.0088	AK8	− 7.50	0.05427	SLC5A8	− 10.26	0.0117
CES1F	− 9.69	0.0113	− 20.50	0.0026	AMY2	− 7.14	0.03483	TNPO1	− 10.19	0.0020
ATP2A3	− 9.18	0.0304	− 16.25	0.0107	DHRS7B	− 7.06	0.01876	POFUT1	− 9.844	0.0022
THBS3	− 9.11	0.0147	− 8.92	0.0196	VAMP5	− 6.65	0.01059	TMC4	− 9.738	0.0027
GAS6	− 9.04	0.0008	− 16.02	0.0002	IGK-V19-17	− 6.56	0.01323	CNTFR	− 9.637	0.0029
FMO1	− 8.95	0.0157	− 9.74	0.0107	FAM213A	− 6.24	0.04285	MCFD2	− 9.555	0.0051
TMX2	− 8.79	0.0440	− 7.70	0.0519	TMED4	− 6.17	0.02456	RSPH4A	− 9.498	0.0141
PIGS	− 8.78	0.0261	− 7.32	0.0318	IDH3B	− 6.00	0.02812	KDSR	− 9.468	0.0137
PGRMC1	− 8.17	0.0021	− 8.46	0.0014	TMCO1	− 5.96	0.03231	SIGMAR1	− 9.407	0.0061
DPM1	− 8.08	0.0141	− 11.59	0.0057	SFXN3	− 5.91	0.01221	ADPGK	− 9.22	0.0197
CDIPT	− 7.93	0.0515	− 11.64	0.0390	ECI1	− 5.79	0.0114	GYS1	− 9.11	0.0142
LYZ2	− 7.53	0.0007	− 11.08	0.0002	FAM160B1	− 5.77	0.00454	SUCLG2	− 8.97	0.0279
CHID1	− 7.42	0.0147	− 17.98	0.0039	SGSH	− 5.77	0.0114	IGKV10-94	− 8.701	0.0111
TMEM205	− 7.21	0.0088	− 8.57	0.0049	ECHS1	− 5.72	0.00802	ABHD16A	− 8.674	0.0183
HACD2	− 7.02	0.0194	− 8.42	0.0107	BC017643	− 5.63	0.02373	DHCR7	− 8.575	0.0146
METTL7A1	− 6.87	0.0053	− 8.58	0.0027	ICA1L	− 5.50	0.00577	EPB4	− 8.257	0.0036
SLC27A4	− 6.79	0.0292	− 13.97	0.0069	UBE2G2	− 5.48	0.03	FKBP2	− 7.817	0.0137
WFDC2	− 6.74	0.0063	− 13.51	0.0011	GTPBP4	− 5.13	0.03997	BPIFB5	− 7.806	0.0051
STT3B	− 6.50	0.0405	− 10.01	0.0158	VKORC1	− 4.90	0.05266	IGHV1-5	− 7.723	0.0183
CES1E	− 6.45	0.0221	− 15.26	0.0035	LRRC37A	− 4.84	0.00822	LMF2	− 7.62	0.0253
FGFR3;FGFR4	− 6.44	0.0014	− 11.70	0.0002	PCDHGC3	− 4.72	0.04258	PRSS1	− 7.527	0.0322
UGT1A6	− 6.38	0.0199	− 9.30	0.0077	IGHV1-9	− 4.66	0.02679	SDF2L1	− 7.512	0.0247
SPCS1	− 6.34	0.0236	− 15.49	0.0035	ERGIC1	− 4.61	0.05083	TLR5	− 7.421	0.0090
ITPR1	− 6.28	0.0530	− 15.03	0.0170	HADHA	− 4.55	0.03026	ACADM	− 7.384	0.0283
TMEM35	− 6.10	0.0121	− 17.46	0.0027	FCN1	− 4.36	0.04129	PRKAR2B	− 7.375	0.0004
TBL2	− 5.75	0.0422	− 10.60	0.0113	DDAH2	− 4.26	0.04271	RPS25	− 7.204	0.0102
CYP2A	− 5.64	0.0014	− 12.45	0.0001	ORM1	− 4.19	0.00759	KTN1	− 7.091	0.0241
SLC4A1	− 5.37	0.0527	− 39.16	0.0025	PTRH2	− 4.14	0.02897	IFITM1	− 7.08	0.0033
LYZ1	− 4.78	0.0012	− 13.95	0.0000	CREG1	− 4.09	0.00591	TAPBP	− 7.005	0.0141
ALG11	− 4.57	0.0527	− 15.11	0.0097	SIRPA	− 3.99	0.01975	SELT	− 6.912	0.0462
CFAP20	− 3.59	0.0230	− 21.17	0.0005	HDHD2	− 3.98	0.02919	LMAN2L	− 6.771	0.0102

Next, a comparison of proteins that were differentially enriched in ozone-exposed males and ozone-exposed females as compared to sex-matched air-exposed mice identified shared and sex-specific signatures. A total of 575 proteins (263, enriched; 312, low abundance) were found differentially expressed in both ozone-exposed males and ozone-exposed females as compared to respective sex-matched air-exposed mice (Fig. 3G, H). As compared to exosomes from air-exposed males, exosomes from ozone-exposed males contained an additional 243 (117, enriched; 126, low abundance) uniquely expressed proteins. As compared to exosomes from air-exposed females, exosomes from ozone-exposed females contained 326 (164, enriched; 162, low abundance) uniquely expressed proteins (Fig. 3G, H).

A list of top shared and sex-specific proteins, relevant to the stress responses, that were found upregulated in ozone-exposed mice is included in Table 3 (Top 50) and Supplemental Table 4 (complete list). Shared protein signatures that were found upregulated in both ozone-exposed males and ozone-exposed females included EPHA2, SLC23A2, SLC26A4, MUC5AC, FN1, POSTN, RETNLA, and various histones (Histone 1, 2, 3). Interestingly, ozone-exposed male mice had significantly upregulated proteins including ITGB6, HSPA2, PTGS2, endophilin-B1 (SH3GLB1), and KEAP1. On the other hand, ozone-exposed female mice had significantly upregulated proteins including PTPN3, APOD, MMP3/MMP10, TSPO, PLA2G7, SH3GL1, and PKP3 (Table 3; Top 50).

A summary of the top 50 proteins that had reduced abundance in ozone-exposed mice are included in Table 4 (Top 50) and Supplemental Table 4 (complete list). Proteins that were found downregulated in both ozone-exposed males and ozone-exposed females included MLF1, LSP1, MERTK, CHID1, THBS3, LYZ2, and FGFR2. Interestingly, ozone-exposed male mice had significantly downregulated proteins including FABP1, AFP, VAMP5, and SGSH. Similarly, ozone-exposed female mice had significantly downregulated proteins including TLR5, PRSS1, LMF1, SLC27A1, and MIA3 (Table 4; Top 50).

### Macrophage activation-associated proteins are differentially enriched in exosomes from ozone-exposed mice

**Figure 4:**
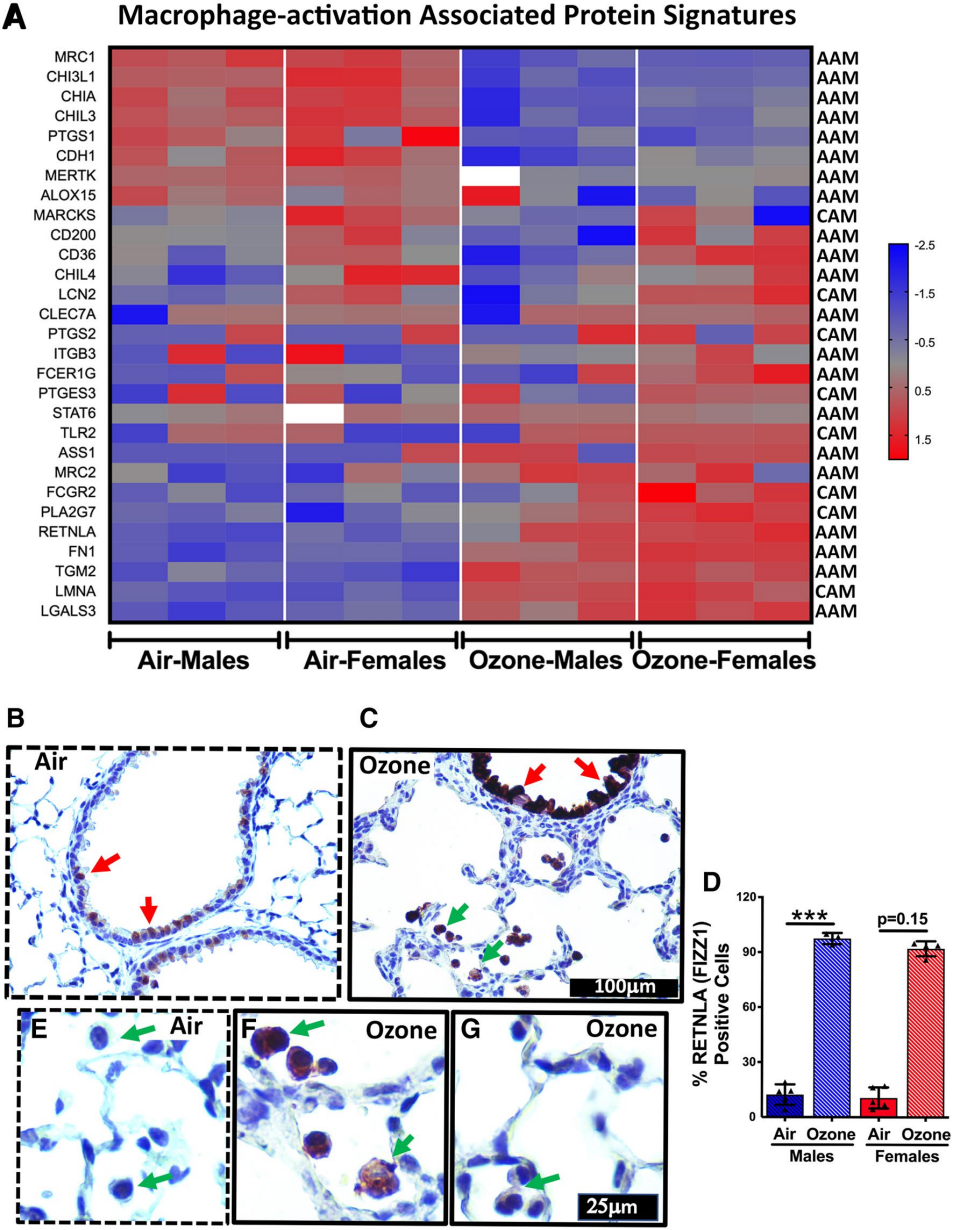
Heat map (**A**) for normalized protein abundance values (Z-scores) representing macrophage activation to classical (CAM, classically-activated macrophages) or alternative (AAM, alternatively-activated macrophages) responses. Higher and lower expressions of each protein are represented by red and blue colors, respectively. (**B-G**) Immunohistochemical analyses of lung sections for cell-specific expression of AAM-associated protein, i.e., RETNLA (FIZZ1). Red arrows point to the RETNLA-stained epithelial cells (**B**, air-exposed; **C**, ozone-exposed). Green arrows point to the macrophages that were positively stained for RETNLA (**C**, ozone-exposed). (**D**) Bar graph showing the proportion of epithelial cells in the small airways that were stained positive for RETNLA. Significant main effect was detected (Kruskal Wallis Statistic = 12.27; df = 3; *p* = 0.0004). Error bars represent Standard Deviation (SD). ***p < 0.001 using Kruskal Wallis test followed by Dunn’s post hoc comparisons (n = 3–5 per group). Green arrows point to the macrophages that remained unstained (**E**, air-exposed; **G**, ozone-exposed;) or those that were intensely stained for RETNLA (**F**, ozone-exposed).

Macrophages within the airspaces have been reported to be activated following ozone exposure^42–44^. Next, through a manual literature search, we prepared a list of proteins that determine the activation status of the macrophages and categorized them into one of the two activation categories, i.e., classically-activated macrophages (CAM, M1) and alternatively-activated macrophages (AAM, M2). Significantly upregulated proteins in exosomes from ozone-exposed mice included LGALS3, TGM2, FN1, LMNA, RETNLA, MRC2, ASS1, and PLA2G7 (Fig. 4A). Significantly downregulated proteins in exosomes from ozone-exposed mice included MRC1, CHI3L1, CHIA, CHI3L3 (YM1), PTGS1, and MERTK. Interestingly, MARCKS, CD200, CD36, LCN2, and CHI3L4 (YM2) were found elevated only in the females, regardless of their treatment groups.

Next, we hypothesized that the macrophage activation protein signatures in the exosomes reflect macrophage activation status following ozone exposure. Our data did not clearly categorize macrophage activation markers into M1 or M2 categories. While overall, a relatively larger number of M2-associated proteins were upregulated in the exosomes from ozone-exposed mice, the protein signatures largely reflected a mixed phenotype. For example, while some M2-associated proteins including RETNLA, TGM2, FN1, MRC2, ASS1, LCN2, and LGALS3 were enriched upon ozone exposure, other M2-associated proteins either remained unchanged (CLEC7A, FCERG1, STAT6) or were present in low abundance (ALOX15, CHIA, CHIL3, CHI3L1, PTGS1, MRC1). Four M2-associated proteins, i.e., CD200, CD36, CHIL4, and LCN2 were specifically upregulated in the females regardless of their exposure status. While some M1-associated markers were enriched (PLA2G7, FCGR2, and LMNA) in the exosomes following ozone exposure, others, such as MARCKS and PTGS2 remained unchanged (Fig. 4A).

RETNLA, commonly known as FIZZ1 (Found in Inflammatory Zone 1), is a well-known marker for alternative macrophage activation, particularly in mice. To determine the cellular source of this protein in the exosomes of ozone-exposed airspaces, we performed immunohistochemical staining on lungs from the air- and ozone-exposed mice (Fig. 4B–G). The majority of the RETNLA-stained cells in air-exposed mice were club cells, however, the staining intensity was very low (Fig. 4B). In contrast, club cells were intensely stained in the ozone-exposed mice (Fig. 4C). In addition to the contrasting staining intensities, an indicator of the differential expression levels of intracellular proteins, the number of RETNLA-stained cells was significantly greater in the ozone-exposed male and trended higher in ozone-exposed female mice (Fig. 4D). Next, we compared RETNLA staining in alveolar macrophages between air- and ozone-exposed mice. The alveolar macrophages did not show any RETNLA staining in air-exposed mice (Fig. 4E). Interestingly, the RETNLA staining in alveolar macrophages from ozone-exposed mice was location-dependent, i.e., intense staining in alveoli adjacent to the terminal bronchioles (Fig. 4F) and no staining in alveoli distal to the terminal bronchioles (Fig. 4G). These findings are consistent with the previous report demonstrating prominent pathological changes in the bronchoalveolar duct regions^45^.

### Mucoinflammatory disease response proteins were differentially enriched in exosomes from ozone-exposed mice

**Figure 5:**
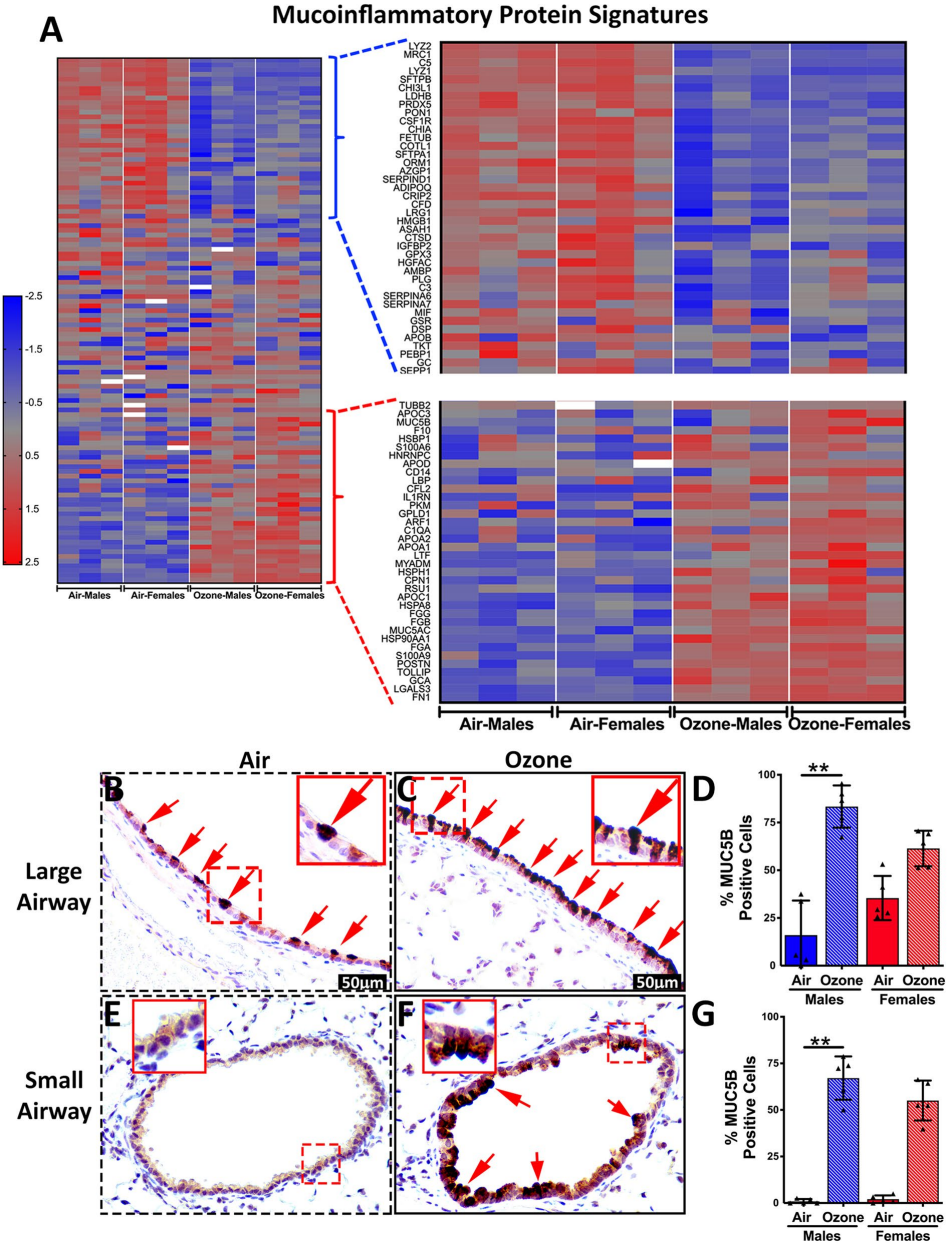
(**A**) Heat maps for normalized values (Z-scores) for proteins associated with mucoinflammatory lung diseases in mice and humans. Low-resolution heat map (Left) depicting expression patterns for the entire muco-inflammatory proteins (High resolution heatmap with protein names is presented as Supplemental Fig. 1). Heat map corresponding to protein signatures that were low abundance (Top) or enriched (Bottom) in exosomes from ozone-exposed mice was amplified for better resolution. (**B**–**G**) Immunohistochemical analyses of lung sections for expression of MUC5B in large airways (**B** and **C**) and small airways (**E** and **F**) from air-exposed (**B** and **E**) and ozone-exposed (**C** and **F**) mice. Significant main effect was detected for both large (Kruskal Wallis Statistic = 15.32; df = 3; *p* = 0.0016) and small (Kruskal Wallis Statistic = 15.17; df = 3; *p* = 0.0017) airways. Red arrows point to the MUC5B-stained epithelial cells (**B**, air-exposed; **C** and **F**, ozone-exposed). Bar graph showing the proportion of epithelial cells in the large (**D**) and small (**G**) airways that were stained positive for MUC5B. Error bars represent Standard Deviation (SD). **p < 0.01 using Kruskal Wallis test followed by Dunn’s post hoc comparisons (n = 5 per group).

Elevated levels of ozone contribute to the exacerbation of pulmonary symptoms in patients with mucoinflammatory lung diseases including asthma^46–48^ and chronic obstructive pulmonary disease^49,50^. Accordingly, through a manual literature search, we customized a list of proteins that have been reported to be associated with mucoinflammatory lung diseases and assessed their levels in the exosomes from the air- and ozone-exposed mice. Ozone exposure resulted in the enrichment of known mucoinflammatory proteins including LGALS3, FN1, POSTN, S100A9, MUC5AC, MUC5B, APOA1, APOA2, FGA, FGB, and FGG (Fig. 5A). Simultaneously, other known mucoinflammatory proteins showed low abundance in exosomes from ozone-exposed mice versus air-exposed mice. These proteins include MRC1, PLG, ADIPOQ, CHIA, LYZ2, CSF1R, SERPIND1, SFTPB, CHI3L1, SFTPA1, LRG1, and HMGB1 (Fig. 5A).

MUC5B is overproduced in the airspaces of patients with mucoinflammatory lung diseases including COPD^51^. To determine the effect of ozone exposure on the intracellular levels of MUC5B in the large versus small airways, we immunohistochemically stained the lung sections and quantified the proportion of cells expressing MUC5B in ozone-exposed versus air-exposed mice (Fig. 5B–G). First-and second-generation airways from air-exposed males and females contained ~ 16% and ~ 35% MUC5B positive epithelial cells, respectively (Fig. 5B, D). In contrast, first-and second-generation airways from ozone-exposed males and females contained ~ 83% and ~ 61% MUC5B positive epithelial cells, respectively (Fig. 5C, D). The preterminal and terminal bronchioles from air-exposed males and females had only ~ 2% and ~ 1% MUC5B positive epithelial cells, respectively (Fig. 5E, G). On the other hand, the preterminal and terminal bronchioles from ozone-exposed males and females had ~ 55% and ~ 67% MUC5B positive epithelial cells, respectively (Fig. 5F, G). These data suggest that the enrichment of MUC5B in the exosomal fraction from ozone-exposed mice is a result of the overproduction of this protein in both large and small airways.

### BALF exosomes carry proteins associated with homeostatic and perturbed lung environment

**Figure 6:**
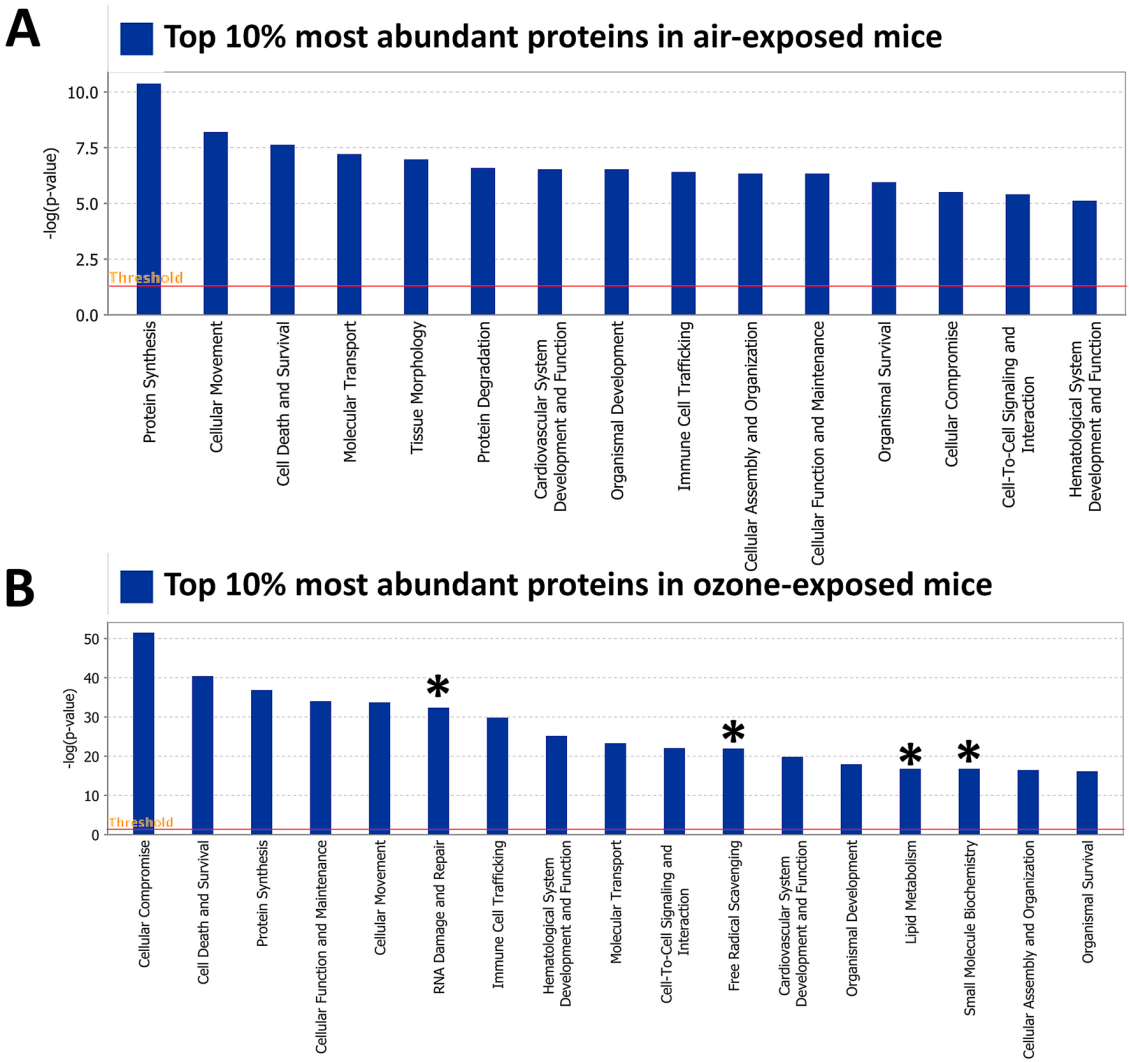
Biological pathway analyses on abundant proteins in BALF exosomes from air- and ozone-exposed mice. (**A**) Ingenuity pathway analysis (IPA) on most abundant (top 10%; 326 out of 3258 identified proteins, determined by Log2 VSN normalized iBAQ MS1 intensities) proteins in exosomes harvested from air-exposed mice. Pathways related to “molecular and cellular functions” and “Physiological system development and function” categories were interrogated for their enrichment. (**B**) Ingenuity pathway analysis (IPA) on most abundant (top 10%; 342 out of 3421 identified proteins, determined by Log2 VSN normalized iBAQ MS1 intensities) proteins in exosomes harvested from ozone-exposed mice. The asterisk represents pathways that were uniquely enriched in exosomes harvested from ozone-exposed mice.

To identify the signaling pathways that are enriched within the exosomal proteins from air-exposed mice, we performed Ingenuity pathway analysis (IPA) on the most abundant (top 10%; 326 out of 3258 identified proteins) proteins. The abundance of proteins was determined by Log2 VSN normalized iBAQ MS1 intensities, i.e., proteins with the highest intensity values were considered most abundant. We searched enrichment of pathways related to “molecular and cellular functions” and “Physiological system development and function” categories. Our analyses identified a number of pathways including protein synthesis, cellular movement, cell death and survival, molecular transport, tissue morphology, protein degradation, organismal development, immune cell trafficking, cellular assembly and organization, cellular function and maintenance, cellular compromise, cell–cell signaling and interaction, and hematological system development and function (Fig. 6A). Similarly, we performed IPA on the most abundant (top 10%; 342 out of 3421 identified proteins) proteins in exosomes harvested from ozone-exposed mice. While most of the pathways, except for tissue morphology and protein degradation, that were enriched in the air-exposed exosomes were also enriched in ozone-exposed exosomes, additional pathways identified in ozone-exposed exosomes were RNA damage and repair, free-radical scavenging, lipid metabolism, and small molecule biochemistry (Fig. 6B). These data show that BALF exosomes carry signatures of the homeostatic lung environment as well as the diseased state.

### Comparative analysis of proteins in BALF exosomes from ozone- vs air-exposed mice reveals enrichment of inflammation/injury-associated pathways and protein interaction networks

**Figure 7:**
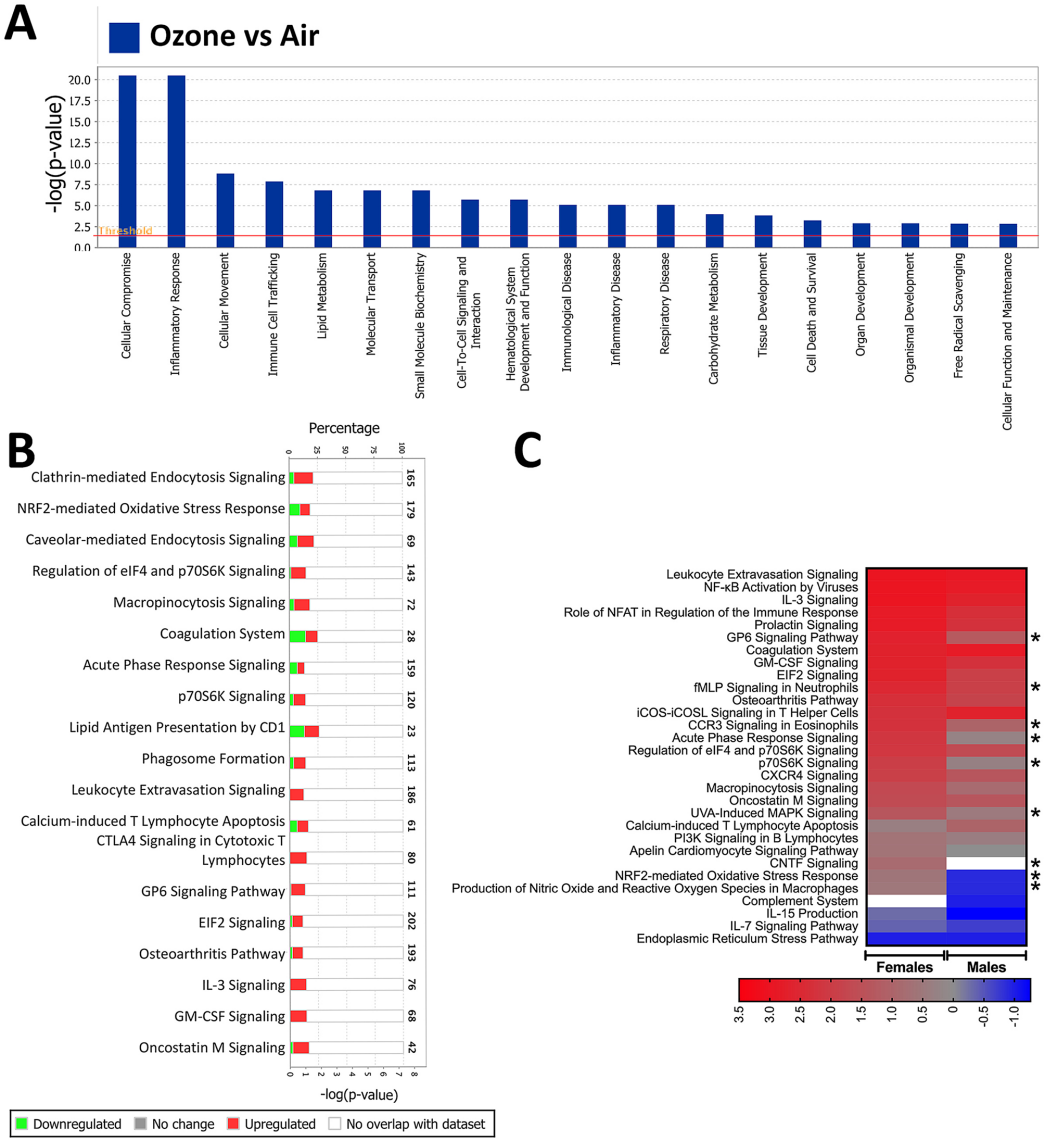
Comparative analysis of proteins in BALF exosomes from ozone- vs air-exposed mice reveal enrichment of inflammation/injury associated pathways. 1255 differentially expressed proteins (Total,1255; enriched, 568; low abundance, 687) were subjected to Ingenuity pathway analysis (IPA). Ingenuity pathway analysis (IPA) for the enrichment of (**A**) disease/functional pathways and (**B**) canonical pathways/biological networks altered in the exosomes from ozone-exposed mice. (**C**) IPA was performed to compare differentially expressed signatures in ozone-exposed males (versus air-exposed males; 380, enriched; 438, low abundance) and ozone-exposed females (versus air-exposed females; 427, enriched; 474, low abundance). Z-scores were used to plot heat maps. Only selected pathways are presented in this figure panel. A detailed heat map with all the differentially enriched pathways is included in Supplemental Fig. 2.

Next, we analyzed disease-associated and functional pathways altered in the exosomes from ozone-exposed mice. Top enriched pathways included cellular compromise, inflammatory responses, cellular movement, immune cell trafficking, lipid metabolism, molecular transport, small molecular biochemistry, cell–cell interaction, hematological system development and function, immunological diseases, inflammatory diseases, respiratory diseases, cell death and survival, and free radical scavenging (Fig. 7A).

To determine the canonical pathways that are associated with the proteins that are enriched in the exosomes of ozone-exposed mice, we subjected differentially (FC > 2, FDR < 0.05) expressed proteins (total, 1255; enriched, 568; low abundance, 687) in exosomes from ozone-exposed mice to pathway analysis using IPA application. Of the top 14 pathways, 9 were upregulated that included micropinocytosis, coagulation system, acute phase response, lipid antigen presentation by CD1, phagosome formation, leukocyte extravasation, calcium-induced T lymphocyte apoptosis, CTLA4 signaling in cytotoxic T lymphocytes, IL-3 signaling, GM-CSF signaling, and Oncostatin M signaling (Fig. 7B). Further, to identify the sex-dependent enrichment of canonical pathways, we performed a comparative analysis between differentially expressed signatures in ozone-exposed males (versus air-exposed males; 380, enriched; 438, low abundance) and ozone-exposed females (versus air-exposed females; 427, enriched; 474, low abundance). The analyses revealed a relatively higher z-score for the majority of pathways in ozone-exposed females versus ozone-exposed males (Fig. 7C and Supplemental Fig. 2). Together, these analyses indicate ozone-induced stress within the airspaces that is reflected within the proteomic signatures of the exosomes present within these airspaces.

Next, to identify enriched protein–protein interaction (PPI) networks, we performed STRING analysis on 568 proteins that were enriched in ozone-exposed mice versus air-exposed mice. The significantly influenced PPI networks due to enriched exosomal protein signatures in the BALF of ozone-exposed mice versus air-exposed mice included extracellular matrix (ECM) organization, ECM-receptor interaction, cell junction organization, membrane trafficking, and neutrophil degranulation (Supplemental Fig. 3).

### Comparative analysis between enriched BALF exosomes proteins and previously published upregulated gene signatures from ozone-exposed mice

In our recent publication^17^, we profiled transcriptomic changes from three distinct lung compartments from ozone-exposed mice. To determine whether the enriched protein signatures reflect upregulation of their transcripts, and to identify the potential source of exosomes carrying these proteins, we interrogated a list of significantly enriched exosomal proteins (568 identified proteins; FC > 2; adj *p*-value < 0.05) against the transcriptome from the airways, parenchyma, and macrophages. Each protein signature was used to extract fold-change and adj *p*-values from the three lung compartments. Transcriptomic signatures meeting cutoff criteria (FC > 1; adj *p*-value < 0.05) from each tissue were considered significantly upregulated. We categorized the compartment-specific association of transcripts with exosomal protein signatures, based on the comparative analyses of gene expression changes across the three lung compartments.

Transcripts encoding 180 enriched protein signatures were upregulated in all three compartments (Table 5). The enrichment of 47 proteins was reflected in the upregulated DEGs from airways. Similarly, transcripts for 38 and 59 enriched proteins in the BALF exosomes were differentially upregulated exclusively in the parenchyma and the alveolar macrophages, respectively. Transcripts for 66 enriched proteins in the BALF exosomes were differentially upregulated in both airways and parenchyma. Similarly, transcripts for 44 and 63 enriched proteins in the BALF exosomes were differentially upregulated in extrapulmonary airways as well as airspace macrophages and parenchyma as well as alveolar macrophages, respectively. Finally, the enrichment of 71 proteins in the BALF exosomes did not reflect upregulated transcripts in any of the three compartments.

**Table 5:** Comparative analyses of enriched protein signatures from ozone-exposed mice and transcriptome from three lung compartments.

Gene signatures upregulated in Airways, Parenchyma, or Alveolar macrophages (FC > 1; adj *p*-value < 0.05)	Number of enriched exosomal proteins (ozone vs air) (FC > 2; adj *p*-value < 0.05)	Selected protein signatures
Airways Parenchyma Alveolar macrophages	180	EPHA2, ITGA3, RETNLA, SLC26A4, MUC5AC, S100A16, SFTPC, F3, ITGB4, TSPAN8, FBL, ADAM10, AGER, CLCA1, S100A14, TGM2, CLEC7A, FGG, MUC4, TFRC, TSPO, LRP2, ANXA4, ANXA1, CLDN18, ADAM9, LDLR
Airways	47	ITGA6, ITGB1, ATP2B1, CKAP5, HGS, DNAJB4,
Parenchyma	38	CD151, ABCA3, ATP9A, FGA, APOC1, TLR2, TGFBR2, HIST1H1E
Alveolar macrophages	59	S100A9, TIMP3, PLA2G7, CD81, ANXA11, MACF1, TMEM2, H2-AB1, CAV1, PTGFRN, BMPR1A
Airways Parenchyma	66	LMNA, H1F0, SAA1, TGM1, LGALS3, LGL2, HIST1H3B, APOD, LTF, A2M, IL1RN, S100A10, S100A11, EPCAM, SLC44A2
Airways Alveolar macrophages	44	TOP1, TSPAN2, TSPAN15, TNC, FCGR2B, CEACAM1, TOLLIP, CPNE8, ECM1, FLNB, CAPN7
Parenchyma Alveolar macrophages	63	ITGAV, COL6A1-3, TNC, SLC23A2, ITGF3, ITGA9, MMP3, COL4A2, KEAP1, C1QC, COL12A1, SLC16A1
None	71	POSTN, NGP, HIST1G2BR, ARHGEF2, CLDN1, AGO2, ANXA6, FGB

568 protein signatures that were found enriched in ozone-exposed mice versus air-exposed mice were interrogated for their respective gene signature upregulation in three different tissues, i.e., airway, parenchyma, and alveolar macrophages.

## Discussion

Upon encountering abiotic and biotic inhaled insults, the resident lung cells, including epithelial cells and macrophages, orchestrate intercellular communication-dependent coordinated responses to restore airspace homeostasis^52^. Apart from the classical mechanisms for intercellular communication, i.e., direct cell–cell interaction or via soluble mediators, the exosomal cargo biomolecules contribute to the intercellular communication across various physio-pathological conditions^53–55^. Exosomes contain biologically active cargo including proteins, lipids, RNA, and DNA that are known to modulate the functioning of the recipient or target cells^56^. These biomolecular signatures also provide insights into the identity and the well-being of cells that secrete these exosomes. Like most other cell types, lung epithelial cells and alveolar macrophages, are known to release exosomes into their extracellular spaces^3,14,15,55^. In this study, to examine the exosomal protein signatures in the airspaces of healthy as well as ozone-stressed lungs of mice, we hypothesized that repetitive ozone exposure triggers the release of exosome-bound inflammatory proteins from various cells that reflect the mucoobstructive lung disease, and that exosomes from ozone-exposed females possess unique protein signatures that cause exaggerated inflammatory responses. This study identified various sex-dependent as well as sex-independent inflammatory proteins that are relevant to mucoobstructive lung diseases.

Under homeostatic conditions, epithelial cells and macrophages are known to communicate with each other to restrict their proinflammatory characteristics^57,58^. This adaptive phenomenon is critical in effectively curbing the exaggerated immune responses and disruption of the gas exchange function of the respiratory tract. To address our first question, i.e., Which proteins are present in the airspace-derived exosomes from healthy lungs, we analyzed the exosomes harvested from the air-exposed males and females (Table 1). Club cell-specific protein (CCSP; also known as SCGB1A1 or uteroglobin), a product of club cells that possesses anti-inflammatory and immunosuppressive properties^59,60^, was the second most abundant protein present within the exosomes from air-exposed mice. Similarly, alveolar epithelial cell-derived pulmonary surfactant proteins (SFTPA, SFTPB, and SFTPD), also known to be immunosuppressive^61–63^, were enriched within the exosomes from air-exposed mice. Other immunosuppressive proteins, MUC1^64–67^ and MUC5B^68^, products of mucous cells, were also recovered within the exosomes from air-exposed mice (data not shown).

Antioxidant host defense system protects the cells of the respiratory mucosal surfaces from the reactive species generated from the relatively high concentration of inhaled oxygen (~ 150 mmHg at sea level in the conducting airways and ~ 100 mmHg at sea level in the alveolar spaces) at basal levels^69^. Consistent with this, exosomes harvested from the air-exposed mice were enriched in antioxidant-response proteins including peroxiredoxin-6 (PRDX6), superoxide dismutase (SOD1), paraoxonase 1 (PON1), NADPH-cytochrome P450 reductase (POR), Microsomal glutathione S-transferase 1 (MGST1), and Carbonyl reductase [NADPH] 2 (CBR2). These data indicate that exosomes harbor biomolecules critical for antioxidant defense mechanisms under homeostatic conditions. Together, our proteomic data from air-exposed exosomes suggest that, under homeostasis, exosomes carry protein signatures that are involved in anti-inflammatory responses and antioxidant defense.

Interestingly, we found protective protein signatures that were specifically upregulated in the females regardless of their exposure status. CD200 is expressed on the airway epithelial cells and is known to restrain lung macrophages^70^, innate lymphoid cells^71^, and lymphoid cells^72^ via binding to CD200R receptor. A higher level of CD200 in the BALF of air-exposed mice suggests its protective role in the airspaces of females under homeostasis. Of note, the alveolar macrophages from ozone-exposed mice had significantly upregulated expression of *Cd200r*^17^. Lipocalin-2 (LCN2), a neutrophil protein that was also elevated in the air-exposed females, is involved in antibacterial defense^73,74^. These data suggest that female lung airspaces contain protective proteins that likely counter the higher susceptibility of females to inflammatory lung diseases^75–77^.

Exposure to inhaled pollutants is known to induce airspace stress, increase the release rate of exosomes, and alter the composition of the exosomes^78,79^. Consistent with these reports, the total protein yield for exosomes trended higher in ozone-exposed mice versus air-exposed mice. Approximately 16.4% of identified proteins (568 out of 3457) were significantly enriched in the exosomes from ozone-exposed mice versus air-exposed mice (Table 3; Fig. 3F). Apart from this, 4.86% (168 out of 3457) proteins (Histones, S100A8, Elastin, Laminins, FGBP1, MPO, FCGR3, Claudin4, 7, Calpain-6, SEMA3F) were found exclusively in the exosomes from ozone-exposed mice (Supplemental Table 3). Collectively, these data indicate that exposure to ozone not only significantly enhances the enrichment of exosomal proteins that were already present under homeostatic conditions, but also induces the secretion and transport of new proteins within the exosomes.

Ozone, being a highly oxidative gas, is known to cause oxidative stress^80,81^, therefore, an efficient antioxidant system is required to minimize the detrimental effects of reactive oxygen species. Interestingly, however, known antioxidant proteins (EPHX1, SOD1, GSTM1, PRDX6, PON1, MGST1, and POR) were significantly suppressed within the exosomes from ozone-exposed mice. Furthermore, the NRF2-mediated oxidative stress response pathway was also suppressed within the exosomes from ozone-exposed versus air-exposed mice. These findings are consistent with the previous report where antioxidative stress response was active after 1 week of exposure but lost after 3 and 6 weeks of ozone exposure^81^.

While resident alveolar macrophages generally remain quiescent under homeostatic conditions, their functionality is significantly enhanced upon stimulation by the microenvironmental cues that are released by the adjoining cells within the stressed airspaces^82^. Macrophage activation, i.e., enhanced functionality, is a known response to ozone inhalation^42–44,17^. Various macrophage activation markers including arginase 1 (ARG1), NOS2, Galectin 3 (LGALS3), and PTGS2 (COX2) have been reported to be increased in mice following acute exposure to ozone^42,83–85^. Here, we hypothesized that the macrophage activation markers will be enriched within the exosomes harvested from the ozone-exposed airspaces. Our analyses revealed significant enrichment of the alternative activation markers within the exosomes of ozone-exposed mice. For example, Galectin 3 (LGALS3), a carbohydrate-binding lectin, highly expressed in the macrophages and epithelial cells, was upregulated in exosomes from ozone-exposed mice. Galectin 3 expression promotes alternative macrophage activation. TGM2, a member of the transglutaminase family of enzymes, that is known to promote alternative activation of macrophages as well as clearance of apoptotic cells by macrophages (efferocytosis)^86,87^ was also enriched in exosomes from ozone-exposed mice. Of note, TGM2 is a consistent marker of alternative activation of macrophages in humans as well as in mice. Similarly, Resistin-like alpha (RETNLA/FIZZ1), another robust marker of alternative activation of macrophages was upregulated in exosomes from ozone-exposed mice. These data suggest that alternatively activated macrophages from ozone-exposed airspaces release their signature proteins within the exosomes. Interestingly, our histochemical analyses suggest that RETNLA protein expression is dramatically upregulated in ozone-exposed airway epithelial (club) cells as well as macrophages, therefore, it is unclear whether the exosomal RETNLA originates from both or one of the two cell types, i.e., macrophages and airway epithelial cells.

The presence of histones in the BALF has been reported in mice with acute lung injury^88^ and patients with acute respiratory distress syndrome^89^. Extracellular histones are known to act as damage-associated molecular patterns leading to proinflammatory outcomes^90^. Although the mechanisms are unclear, the extracellular histones cause cytotoxic effects on endothelial and epithelial cells^89–91^. Interestingly, BALF from ozone-exposed mice were enriched in histones including HIST1H4A, HIST1H1C, HIST1H2A’s, HIST1H2B’s, and HIST1H3B. While the cellular source of these histones remains unclear, their appearance in the BALF is clearly associated with pathological responses such as granulocyte recruitment, macrophage activation, and airway epithelial remodeling.

Enrichment of certain proteins within the exosomes from stressed airspaces may contribute to the activation of functional and disease pathways. IP analyses on enriched proteins within the exosomes from ozone-exposed mice revealed the activation of several canonical pathways including acute phase response signaling, leucocyte extravasation signaling, production of reactive oxygen species by macrophages, micropinocytosis, phagosome formation, phagosome maturation, lipid antigen presentation, GM-CSF signaling, and IL-3 signaling pathways. These data suggest that the exosomes from ozone-exposed airspaces contain proteins that influence pathways relevant to the inflammatory responses.

Ozone exposure results in the exacerbation of the respiratory symptoms in patients with mucoinflammatory lung diseases such as COPD and asthma^46–48^. Exposure to high levels of ozone for nearly a decade increases the susceptibility to the development of COPD^92^. Similarly, ozone exposure is associated with a decline in lung function and increase in the levels of biomarkers of airway inflammation in asthmatic patients^93^. To observe the effects of ozone exposure in healthy mice and their likelihood of developing hallmarks of mucoinflammatory lung disease, we examined the enrichment of protein markers of mucoobstructive lung diseases within the exosomes from ozone-exposed mice. A large number of proteins that have been previously associated with the mucoobstructive lung diseases were enriched within the exosomes from ozone-exposed mice. These proteins include Galectin 3 (LGALS3), GCA, S100A9, Periostin (POSTN), MUC5AC, MUC5B, FGA, FGB, FGG, FN1, APOC1, IL1RN, and TOLLIP.

Previous reports have demonstrated that female mice show exaggerated inflammatory responses to ozone inhalation as compared to their male counterparts. In our recent report^17^, we also reported that, as compared to ozone-exposed males, ozone-exposed females exhibit exaggerated recruitment of inflammatory cells including macrophages, neutrophils, eosinophils, and lymphocytes. Consistent with cellular recruitment, the levels of total protein trended higher within the lungs of the ozone-exposed females versus ozone-exposed males. Further, the inflammatory mediators including G-CSF, KC, IP-10, IL-6, and IL-5 were significantly elevated in ozone-exposed females versus air-exposed females (Fig. 1). Accordingly, we hypothesized that the exosomes from ozone-exposed females either have significant enrichment of proteins relevant to proinflammatory responses or a significant reduction in the abundance of proteins relevant to anti-inflammatory responses.

Regardless of the comparable amounts of total protein contents within the exosomes of the ozone-exposed male and female mice, exosomes from ozone-exposed females were different from those of the ozone-exposed males in multiple ways. First, although both ozone-exposed sexes shared enrichment of 263 proteins in their exosomes, exosomes from ozone-exposed females had an exclusive enrichment of 164 proteins as compared to 117 in ozone-exposed males (Fig. 3G, H). Second, in addition to 312 proteins that were suppressed upon ozone exposure in both sexes, additional 162 and 126 proteins were exclusively suppressed in ozone-exposed females and ozone-exposed males, respectively. Third, comparative analyses of the canonical pathways between ozone-exposed females and ozone-exposed males revealed higher z-scores in females (Fig. 7C and Supplemental Fig. 2). While these findings point towards an increased enrichment of airspace-relevant stress proteins in ozone-exposed females, the causal-effect relationship between exaggerated inflammatory responses and enrichment of inflammation-relevant exosomal proteins in females remains unexplored.

Identification of the cellular sources of exosomes within the lungs is often challenging because all the resident cells present within the airspaces are known to release exosomes. In exosomes from air-exposed mice, macrophage-specific (CHIL3, LYZ2), airway epithelial cell-specific (SCGB1A1), and alveolar epithelial cell-specific (SFTPA1, SFTPB, SFTPC) proteins were among the most abundant proteins. While these data suggest that, under steady-state, epithelial cells as well as macrophages actively release exosomes into the airspaces, the relative contribution of individual cell types towards the overall exosome populations in the airspaces remains challenging to determine. Proteins including Histones, Annexins, S100s, and RETNLA that were relatively less abundant in exosomes from air-exposed mice were highly abundant in exosomes from ozone-exposed mice suggesting the increased release of these proteins in ozone-exposed airspaces. We hypothesized that the exosomal enrichment of these proteins is contributed by the cellular compartment that overexpresses their respective transcripts. Accordingly, comparative analyses between exosomal proteins and their respective transcripts from the three compartments (airway, parenchyma, macrophages) revealed interesting findings. Transcript levels of the enriched exosomic proteins including histones, RETNLA, Annexins, and S100s were elevated in more than one compartment, if not all three. These data suggest that the ozone stimulates the release of certain proteins from multiple cellular types.

In conclusion, this study reveals various interesting findings. First, under unchallenged conditions, resident cells shed exosomes that contain protein signatures relevant to homeostasis, cell-specificity, and antioxidant defense. Second, ozone exposure contributes to a significant enrichment of those proteins within the exosomes that were present under the homeostatic state. Third, ozone exposure, in addition, stimulates the release of stress-related proteins within the exosomes. Fourth, proteins enriched within the exosomes from ozone-exposed mice represent activation of pathways associated with stress-response. Fifth, comparative analyses of the exosomes from ozone-exposed mice identified sex-specific protein signatures. This is also true for the secretory proteins present within the cell-free BALF following ozone exposure. Sixth, cellular localization of selected muco-inflammatory disease-related proteins revealed the potential cellular contributors of these proteins in the exosomal compartment following ozone exposure. Finally, comparative analyses between the exosomal proteins and the lung compartment-specific transcriptomic signatures revealed compartment-specific contribution towards exosomal protein contents. Collectively, this study presents detailed proteomic analyses of exosomes from homeostatic and ozone-stressed airspaces in mice. These data will aid in future mechanistic studies to unravel underlying inflammation-relevant pathways in ozone-exposed lungs.

## Supplementary Information

Supplementary Tables.

Supplementary Figures.

## References

[1] Colombo M, Raposo G, Thery C (2014). Biogenesis, secretion, and intercellular interactions of exosomes and other extracellular vesicles. Annu. Rev. Cell Dev. Biol..

[2] Thery C, Zitvogel L, Amigorena S (2002). Exosomes: Composition, biogenesis and function. Nat. Rev. Immunol..

[3] McVey MJ, Maishan M, Blokland KEC, Bartlett N, Kuebler WM (2019). Extracellular vesicles in lung health, disease, and therapy. Am. J. Physiol. Lung Cell. Mol. Physiol..

[4] Muller L, Hong CS, Stolz DB, Watkins SC, Whiteside TL (2014). Isolation of biologically-active exosomes from human plasma. J. Immunol. Methods.

[5] Grant R (2011). A filtration-based protocol to isolate human plasma membrane-derived vesicles and exosomes from blood plasma. J. Immunol. Methods.

[6] Paredes PT (2012). Bronchoalveolar lavage fluid exosomes contribute to cytokine and leukotriene production in allergic asthma. Allergy.

[7] Admyre C (2003). Exosomes with major histocompatibility complex class II and co-stimulatory molecules are present in human BAL fluid. Eur. Respir. J..

[8] Pisitkun T, Shen RF, Knepper MA (2004). Identification and proteomic profiling of exosomes in human urine. Proc. Natl. Acad. Sci. U. S. A..

[9] Gonzales PA (2010). Isolation and purification of exosomes in urine. Methods Mol. Biol..

[10] Chanteloup G (2020). Monitoring HSP70 exosomes in cancer patients' follow up: A clinical prospective pilot study. J. Extracell. Vesicles.

[11] Amiri A (2020). Exosomes and Lung cancer: Roles in pathophysiology, diagnosis and therapeutic applications. Curr. Med. Chem..

[12] McVey MJ, Spring CM, Semple JW, Maishan M, Kuebler WM (2016). Microparticles as biomarkers of lung disease: Enumeration in biological fluids using lipid bilayer microspheres. Am. J. Physiol. Lung Cell. Mol. Physiol..

[13] Mathieu M, Martin-Jaular L, Lavieu G, Thery C (2019). Specificities of secretion and uptake of exosomes and other extracellular vesicles for cell-to-cell communication. Nat. Cell Biol..

[14] Wahlund CJE, Eklund A, Grunewald J, Gabrielsson S (2017). Pulmonary extracellular vesicles as mediators of local and systemic inflammation. Front. Cell Dev. Biol..

[15] Andres J (2020). Role of extracellular vesicles in cell-cell communication and inflammation following exposure to pulmonary toxicants. Cytokine Growth Factor Rev..

[16] Lee H, Zhang D, Laskin DL, Jin Y (2018). Functional evidence of pulmonary extracellular vesicles in infectious and noninfectious lung inflammation. J. Immunol..

[17] Choudhary I, Vo T, Paudel K, Patial S, Saini Y (2021). Compartment-specific transcriptomics of ozone-exposed murine lungs reveals sex- and cell type-associated perturbations relevant to mucoinflammatory lung diseases. Am. J. Physiol. Lung Cell. Mol. Physiol..

[18] Harkema JR (2017). Strain differences in a murine model of air pollutant-induced nonatopic asthma and rhinitis. Toxicol. Pathol..

[19] Hatch GE (2014). Progress in assessing air pollutant risks from in vitro exposures: Matching ozone dose and effect in human airway cells. Toxicol. Sci..

[20] Hatch GE (2013). Biomarkers of dose and effect of inhaled ozone in resting versus exercising human subjects: Comparison with resting rats. Biomark. Insights.

[21] Hatch GE (1994). Ozone dose and effect in humans and rats. A comparison using oxygen-18 labeling and bronchoalveolar lavage. Am. J. Respir. Crit. Care Med..

[22] Cabello N (2015). Sex differences in the expression of lung inflammatory mediators in response to ozone. Am. J. Physiol.-Lung Cell. Mol. Physiol..

[23] Birukova A (2019). Sex modifies acute ozone-mediated airway physiologic responses. Toxicol. Sci..

[24] Tashiro H (2020). Sex differences in the impact of dietary fiber on pulmonary responses to ozone. Am. J. Respir. Cell Mol. Biol..

[25] Cho Y (2019). Sex differences in pulmonary responses to ozone in mice. Role of the microbiome. Am. J. Respir. Cell Mol. Biol..

[26] Peirson SN, Foster RG (2011). Bad light stops play. EMBO Rep..

[27] Thery C, Amigorena S, Raposo G, Clayton A (2006). Isolation and characterization of exosomes from cell culture supernatants and biological fluids. Curr. Protoc. Cell Biol..

[28] Nesvizhskii AI, Keller A, Kolker E, Aebersold R (2003). A statistical model for identifying proteins by tandem mass spectrometry. Anal. Chem..

[29] Huber W, von Heydebreck A, Sultmann H, Poustka A, Vingron M (2002). Variance stabilization applied to microarray data calibration and to the quantification of differential expression. Bioinformatics.

[30] Bolstad, B. M. Preprocess core: A collection of pre-processing functions. *R package version 1.48.0.* (2019).

[31] Ritchie ME (2015). limma powers differential expression analyses for RNA-sequencing and microarray studies. Nucl. Acids Res..

[32] Chawade A, Alexandersson E, Levander F (2014). Normalyzer: A tool for rapid evaluation of normalization methods for omics data sets. J. Proteome Res..

[33] Alhamdoosh M (2017). Easy and efficient ensemble gene set testing with EGSEA. F1000Res.

[34] Szklarczyk D (2019). STRING v11: Protein-protein association networks with increased coverage, supporting functional discovery in genome-wide experimental datasets. Nucl. Acids Res..

[35] Lewis BW (2020). Ablation of IL-33 suppresses Th2 responses but is accompanied by sustained mucus obstruction in the Scnn1b transgenic mouse model. J. Immunol..

[36] Lewis BW (2020). The innate lymphoid system is a critical player in the manifestation of mucoinflammatory airway disease in mice. J. Immunol..

[37] Nadadur SS, Costa DL, Slade R, Silbjoris R, Hatch GE (2005). Acute ozone-induced differential gene expression profiles in rat lung. Environ. Health Perspect..

[38] Hu PC (1982). Protein accumulation in lung lavage fluid following ozone exposure. Environ. Res..

[39] Joyner BL (2013). DNA and inflammatory mediators in bronchoalveolar lavage fluid from children with acute inhalational injuries. J. Burn Care Res..

[40] Kirchner KK, Wagener JS, Khan TZ, Copenhaver SC, Accurso FJ (1996). Increased DNA levels in bronchoalveolar lavage fluid obtained from infants with cystic fibrosis. Am. J. Respir. Crit. Care Med..

[41] Choudhary, I. *et al.* Postnatal ozone exposure disrupts alveolar development, exaggerates mucoinflammatory responses, and suppresses bacterial clearance in developing Scnn1b-Tg+ mouse lungs. *J. Immunol. *accepted (2021). 10.4049/jimmunol.2001286 PMC8654340 34330754

[42] Sunil VR, Patel-Vayas K, Shen J, Laskin JD, Laskin DL (2012). Classical and alternative macrophage activation in the lung following ozone-induced oxidative stress. Toxicol. Appl. Pharmacol..

[43] Groves AM (2013). Age-related increases in ozone-induced injury and altered pulmonary mechanics in mice with progressive lung inflammation. Am. J. Physiol. Lung Cell. Mol. Physiol..

[44] Mathews JA (2015). Gammadelta T cells are required for M2 macrophage polarization and resolution of ozone-induced pulmonary inflammation in mice. PLoS ONE.

[45] Pinkerton, K. E., Menache, M. G. & Plopper, C. G. Consequences of prolonged inhalation of ozone on F344/N rats: Collaborative studies. Part IX: Changes in the tracheobronchial epithelium, pulmonary acinus, and lung antioxidant enzyme activity. *Res. Rep. Health Eff. Inst*. 41–98; discussion 99–110 (1995). 7619334

[46] McDonnell WF, Abbey DE, Nishino N, Lebowitz MD (1999). Long-term ambient ozone concentration and the incidence of asthma in nonsmoking adults: The AHSMOG Study. Environ. Res..

[47] Anenberg SC (2018). Estimates of the global burden of ambient [formula: see text], ozone, and [formula: see text] on asthma incidence and emergency room visits. Environ. Health Perspect..

[48] Tetreault LF (2016). Childhood exposure to ambient air pollutants and the onset of asthma: An administrative cohort study in Quebec. Environ. Health Perspect..

[49] Strosnider HM (2019). Age-specific associations of ozone and fine particulate matter with respiratory emergency department visits in the United States. Am. J. Respir. Crit. Care Med..

[50] Paulin LM, Kaufman JD, Hansel NN (2020). Concerns remain regarding long-term ozone exposure and respiratory outcomes-reply. JAMA Intern. Med..

[51] Kirkham S (2008). MUC5B is the major mucin in the gel phase of sputum in chronic obstructive pulmonary disease. Am. J. Respir. Crit. Care Med..

[52] Boitano S, Safdar Z, Welsh DG, Bhattacharya J, Koval M (2004). Cell-cell interactions in regulating lung function. Am. J. Physiol. Lung Cell. Mol. Physiol..

[53] Gupta R (2019). Intercellular communication between airway epithelial cells is mediated by exosome-like vesicles. Am. J. Respir. Cell Mol. Biol..

[54] Lee H, Abston E, Zhang D, Rai A, Jin Y (2018). Extracellular vesicle: An emerging mediator of intercellular crosstalk in lung inflammation and injury. Front. Immunol..

[55] Moon HG (2015). Lung epithelial cell-derived extracellular vesicles activate macrophage-mediated inflammatory responses via ROCK1 pathway. Cell Death Dis..

[56] Yanez-Mo M (2015). Biological properties of extracellular vesicles and their physiological functions. J. Extracell. Vesicles.

[57] Haggadone MD, Peters-Golden M (2018). Microenvironmental influences on extracellular vesicle-mediated communication in the lung. Trends Mol. Med..

[58] Fujita Y, Kosaka N, Araya J, Kuwano K, Ochiya T (2015). Extracellular vesicles in lung microenvironment and pathogenesis. Trends Mol. Med..

[59] Levin SW, Butler JD, Schumacher UK, Wightman PD, Mukherjee AB (1986). Uteroglobin inhibits phospholipase A2 activity. Life Sci..

[60] Hayashida S, Harrod KS, Whitsett JA (2000). Regulation and function of CCSP during pulmonary Pseudomonas aeruginosa infection in vivo. Am. J. Physiol. Lung Cell. Mol. Physiol..

[61] Rosseau S (1999). Surfactant protein A down-regulates proinflammatory cytokine production evoked by Candida albicans in human alveolar macrophages and monocytes. J. Immunol..

[62] Ikegami M (2007). Surfactant protein-D and surfactant inhibit endotoxin-induced pulmonary inflammation. Chest.

[63] Allen JN, Moore SA, Pope-Harman AL, Marsh CB, Wewers MD (1995). Immunosuppressive properties of surfactant and plasma on alveolar macrophages. J. Lab. Clin. Med..

[64] Ueno K (2008). MUC1 mucin is a negative regulator of toll-like receptor signaling. Am. J. Respir. Cell. Mol. Biol..

[65] Sheng YH (2013). MUC1 and MUC13 differentially regulate epithelial inflammation in response to inflammatory and infectious stimuli. Mucosal Immunol..

[66] Li Y, Dinwiddie DL, Harrod KS, Jiang Y, Kim KC (2010). Anti-inflammatory effect of MUC1 during respiratory syncytial virus infection of lung epithelial cells in vitro. Am. J. Physiol. Lung Cell. Mol. Physiol..

[67] Guang W (2010). Muc1 cell surface mucin attenuates epithelial inflammation in response to a common mucosal pathogen. J. Biol. Chem..

[68] Roy MG (2014). Muc5b is required for airway defence. Nature.

[69] Cantin AM, Fells GA, Hubbard RC, Crystal RG (1990). Antioxidant macromolecules in the epithelial lining fluid of the normal human lower respiratory tract. J. Clin. Invest..

[70] Snelgrove RJ (2008). A critical function for CD200 in lung immune homeostasis and the severity of influenza infection. Nat. Immunol..

[71] Shafiei-Jahani P (2021). CD200-CD200R immune checkpoint engagement regulates ILC2 effector function and ameliorates lung inflammation in asthma. Nat. Commun..

[72] Rijkers ES (2008). The inhibitory CD200R is differentially expressed on human and mouse T and B lymphocytes. Mol. Immunol..

[73] Berger T (2006). Lipocalin 2-deficient mice exhibit increased sensitivity to *Escherichia coli* infection but not to ischemia-reperfusion injury. Proc. Natl. Acad. Sci. U. S. A..

[74] Bachman MA, Miller VL, Weiser JN (2009). Mucosal lipocalin 2 has pro-inflammatory and iron-sequestering effects in response to bacterial enterobactin. PLoS Pathog..

[75] Barnes PJ (2016). Sex differences in chronic obstructive pulmonary disease mechanisms. Am. J. Respir. Crit. Care Med..

[76] Kokturk N, Kilic H, Baha A, Lee SD, Jones PW (2016). Sex difference in chronic obstructive lung disease. Does it matter? A concise review. COPD.

[77] Tam A (2016). Sex differences in airway remodeling in a mouse model of chronic obstructive pulmonary disease. Am. J. Respir. Crit. Care Med..

[78] Lee H, Zhang D, Wu J, Otterbein LE, Jin Y (2017). Lung epithelial cell-derived microvesicles regulate macrophage migration via microRNA-17/221-induced integrin beta1 recycling. J. Immunol..

[79] Bourdonnay E (2015). Transcellular delivery of vesicular SOCS proteins from macrophages to epithelial cells blunts inflammatory signaling. J. Exp. Med..

[80] Mudway IS (1999). Compromised concentrations of ascorbate in fluid lining the respiratory tract in human subjects after exposure to ozone. Occup. Environ. Med..

[81] Wiegman CH (2014). A comprehensive analysis of oxidative stress in the ozone-induced lung inflammation mouse model. Clin. Sci. (Lond.).

[82] Mosser DM, Edwards JP (2008). Exploring the full spectrum of macrophage activation. Nat. Rev. Immunol..

[83] Sunil VR (2015). Regulation of ozone-induced lung inflammation and injury by the beta-galactoside-binding lectin galectin-3. Toxicol. Appl. Pharmacol..

[84] Sunil VR (2013). Ozone-induced injury and oxidative stress in bronchiolar epithelium are associated with altered pulmonary mechanics. Toxicol. Sci..

[85] Cabello N (2015). Sex differences in the expression of lung inflammatory mediators in response to ozone. Am. J. Physiol. Lung Cell. Mol. Physiol..

[86] Martinez FO (2013). Genetic programs expressed in resting and IL-4 alternatively activated mouse and human macrophages: Similarities and differences. Blood.

[87] Eligini S, Fiorelli S, Tremoli E, Colli S (2016). Inhibition of transglutaminase 2 reduces efferocytosis in human macrophages: Role of CD14 and SR-AI receptors. Nutr. Metab. Cardiovasc. Dis..

[88] Yue X, Guidry JJ (2019). Differential protein expression profiles of bronchoalveolar lavage fluid following lipopolysaccharide-induced direct and indirect lung injury in mice. Int. J. Mol. Sci..

[89] Bosmann M (2013). Extracellular histones are essential effectors of C5aR- and C5L2-mediated tissue damage and inflammation in acute lung injury. FASEB J..

[90] Huang H (2011). Endogenous histones function as alarmins in sterile inflammatory liver injury through toll-like receptor 9 in mice. Hepatology.

[91] Xu J (2009). Extracellular histones are major mediators of death in sepsis. Nat. Med..

[92] Paulin LM (2019). Association of long-term ambient ozone exposure with respiratory morbidity in smokers. JAMA Intern. Med..

[93] Khatri SB (2009). Association of ambient ozone exposure with airway inflammation and allergy in adults with asthma. J. Asthma.

