# Supplementary material for: Vesicular and extravesicular protein analyses from the airspaces of ozone-exposed mice revealed signatures associated with mucoinflammatory lung disease
Source: Sci Rep. 2021 Dec 1;11:23203. doi: 10.1038/s41598-021-02256-5 (PMC8636509; doi:10.1038/s41598-021-02256-5)
Supplement: Supplementary file 2 — Supplementary Figures. [file 41598_2021_2256_MOESM2_ESM.pdf]

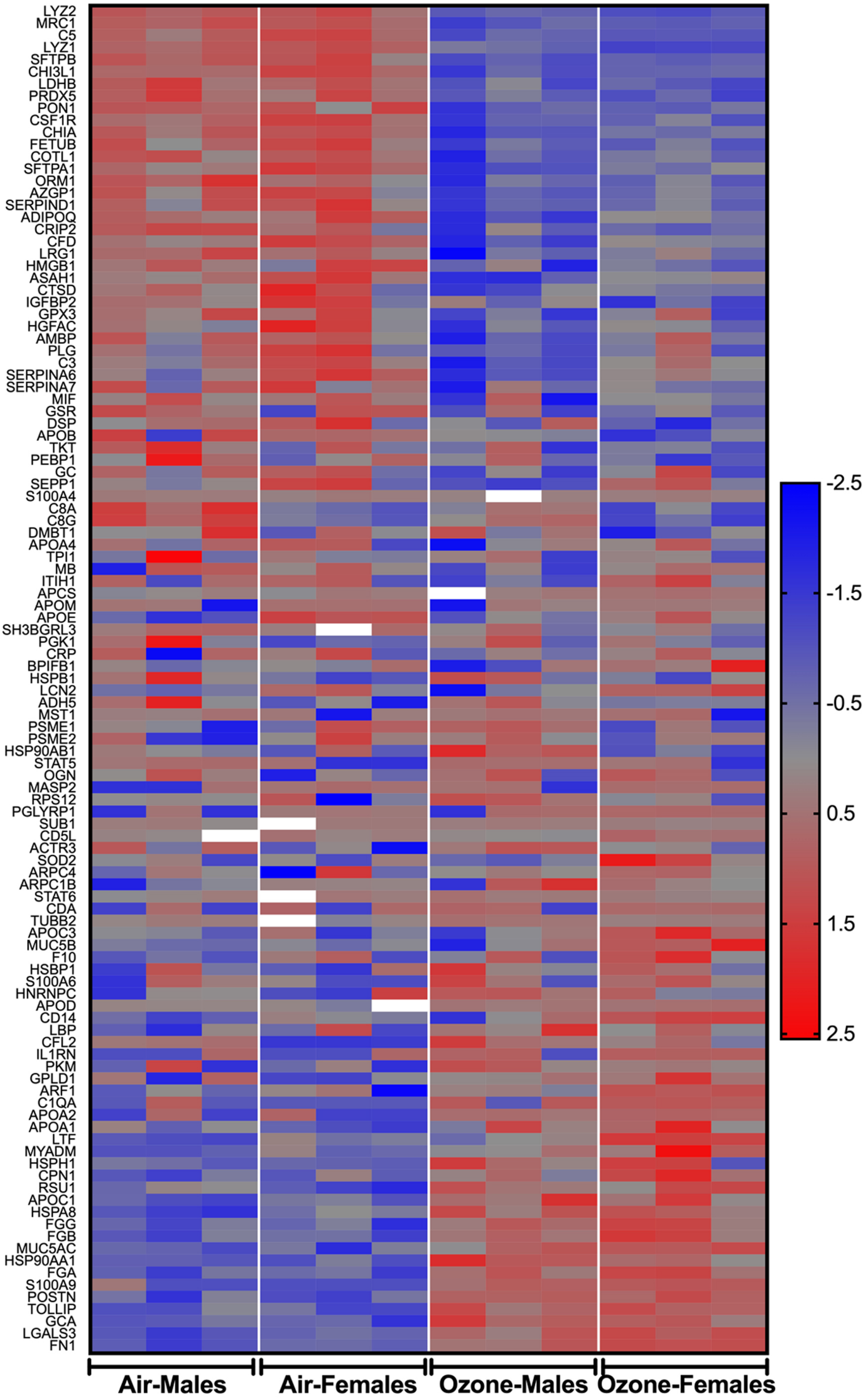

Supplemental Figure 1

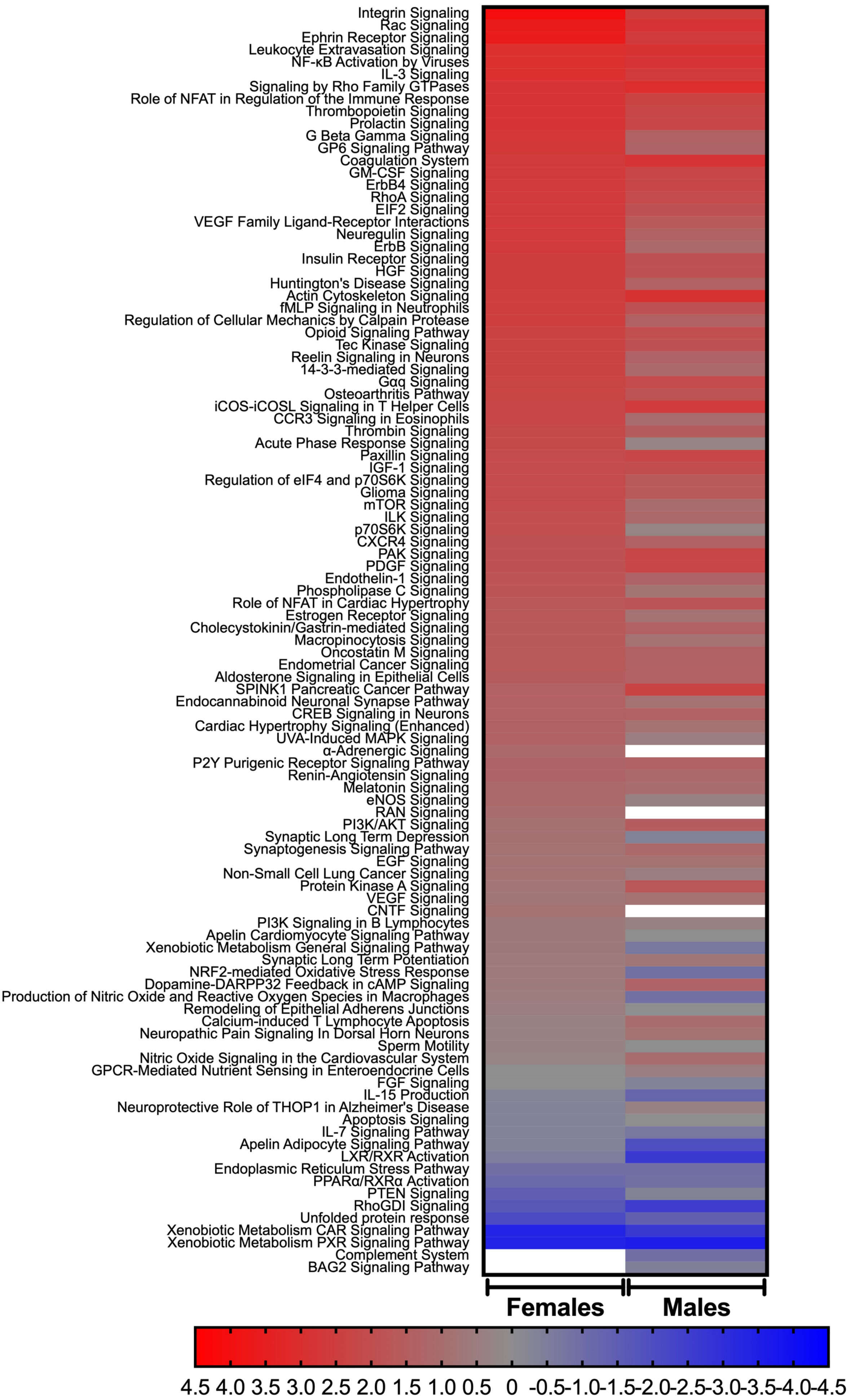

Supplemental Figure 2

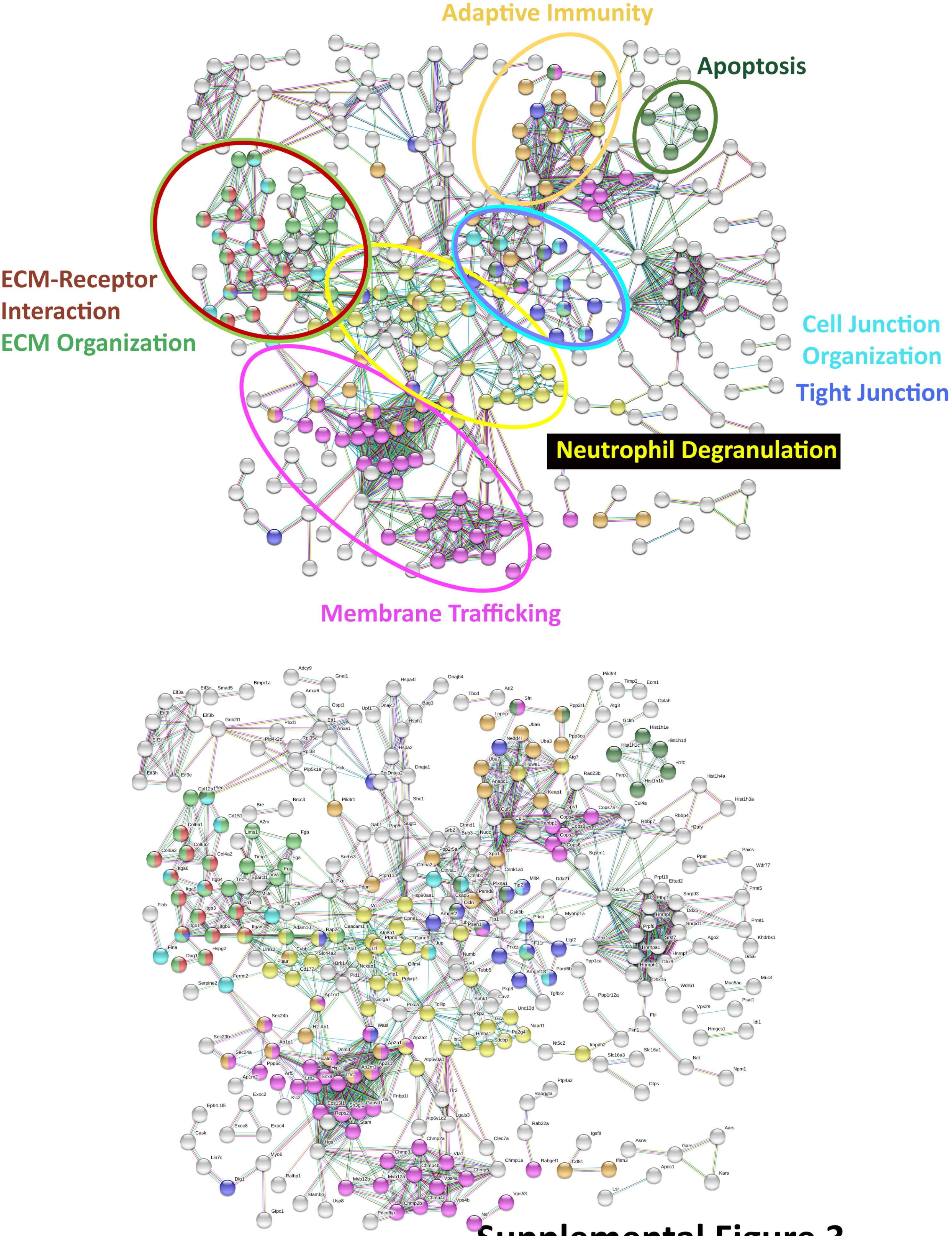

Supplemental Figure 3

Supplemental Figures:

Supplemental Fig. 1: High-resolution heatmap (supporting image for **Figure 5A**) for normalized expression values (Z-scores) of protein signatures associated with mucoinflammatory lung diseases in mice and humans.

Supplemental Fig. 2: IPA was performed to compare differentially expressed signatures in ozone-exposed males (versus air-exposed males; 380, enriched; 438, low-abundance) and ozone-exposed females (versus air-exposed females; 427, enriched; 474, low-abundance). Z-scores were used to plot heat maps.

Supplemental Fig. 3: STRING database protein-protein interaction network analyses on enriched proteins (568) in exosomes from ozone-exposed mice versus air-exposed mice. Interactions were determined based on evidence, using the highest confidence level (0.9) setting. 547 nodes and 1052 edges were identified. Disconnected nodes were selected to be hidden. (PPI enrichment p-value<1.0 $X10^{-16}$). Proteins involved in ECM-receptor interaction (Counts=14/81; FDR adjusted p-value<2.79 $X10^{-06}$), Tight Junction (Counts=18/165; FDR adjusted p-value<1.32 $X10^{-5}$), Membrane trafficking (Counts=47/523; FDR adjusted p-value<5.5 $X10^{-11}$), Extracellular matrix organization (Counts=31/246; FDR adjusted p-value<2.53 $X10^{-10}$), Cell junction organization (Counts=16/63; FDR adjusted p-value<5.07 $X10^{-09}$), Neutrophil degranulation (Counts=39/476; FDR adjusted p-value<2.62 $X10^{-08}$), Apoptosis (Counts=13/93; FDR adjusted p-value<6.28 $X10^{-05}$), and Adaptive immune system (Counts=34/652; FDR adjusted p-value<0.0014) were encircled.
